# Addendum: Cruz, B., et al. Leucine-Rich Diet Modulates the Metabolomic and Proteomic Profile of Skeletal Muscle during Cancer Cachexia. *Cancers* **2020**, *12*, 1880

**DOI:** 10.3390/cancers13040880

**Published:** 2021-02-20

**Authors:** Bread Cruz, André Oliveira, Lais Rosa Viana, Leisa Lopes-Aguiar, Rafael Canevarolo, Maiara Caroline Colombera, Rafael Rossi Valentim, Fernanda Garcia-Fóssa, Lizandra Maia de Sousa, Bianca Gazieri Castelucci, Sílvio Roberto Consonni, Daniel Martins-de-Souza, Marcelo Bispo de Jesus, Steven Thomas Russell, Maria Cristina C. Gomes-Marcondes

**Affiliations:** 1 Department Structural and Functional Biology, Institute of Biology, UNICAMP, Campinas 13083-970, SP, Brazil; bread.cruz@gmail.com (B.C.); ago_oliveira@yahoo.com (A.O.); lala.viana311088@gmail.com (L.R.V.); leisaaguiar@yahoo.com.br (L.L.-A.); colomberamaiara@gmail.com (M.C.C.); rafaelrossiphd@gmail.com (R.R.V.); 2 Department Cancer Physiology, H. Lee Moffitt Cancer Center and Research Institute, Tampa, FL 33612, USA; rafaelcanevarolo@gmail.com; 3 Nano-Cell Interactions Lab., Department Biochemistry and Tissue Biology, Institute of Biology, UNICAMP, Campinas 13083-970, SP, Brazil; fefossa@gmail.com (F.G.-F.); dejesus@unicamp.br (M.B.d.J.); 4 Biochemistry and Tissue Biology, Institute of Biology, UNICAMP, Campinas 13083-970, SP, Brazil; l172421@dac.unicamp.br (L.M.d.S.); biancastelucci@gmail.com (B.G.C.); consonni@unicamp.br (S.R.C.); 5 Laboratory of Neuroproteomics, Department of Biochemistry and Tissue Biology, Institute of Biology, University of Campinas, Campinas 13083-970, SP, Brazil; dmsouza@unicamp.br; 6 Experimental Medicine Research Cluster (EMRC), University of Campinas, Campinas 13083-970, SP, Brazil; 7 D’Or Institute for Research and Education (IDOR), Rio de Janeiro 04501-000, SP, Brazil; 8 Biology and Biomedical Sciences, School of Life and Health Sciences, Aston University, Birmingham B4 7ET, UK; s.t.russell1@aston.ac.uk

The authors wish to add the following statement in the Acknowledgements section of this paper [1]:

The authors would like to acknowledge Dr. Natália Tobar, Division of Nuclear Medicine, Department of Radiology, School of Medical Sciences, University of Campinas, for the excellent acquisition and discussion of animal parameters by DEXA.

The authors apologize for any inconvenience caused and state that the scientific conclusions are unaffected.
