# Supplementary material for: Leucine-Rich Diet Modulates the Metabolomic and Proteomic Profile of Skeletal Muscle during Cancer Cachexia
Source: Cancers (Basel). 2020 Jul 13;12(7):1880. doi: 10.3390/cancers12071880 (PMC7408981; doi:10.3390/cancers12071880)
Supplement: Supplementary file 1 [file cancers-12-01880-s001.pdf]

Supplementary Materials

Leucine-Rich Diet Modulates the Metabolomic and Proteomic Profile of Skeletal Muscle during Cancer Cachexia

Bread Cruz, André Oliveira, Lais Rosa Viana, Leisa Lopes-Aguiar, Rafael Canevarolo, Maiara Caroline Colombera, Rafael Rossi Valentim, Fernanda Garcia-Fóssa, Lizandra Maia de Sousa, Bianca Gazieri Castelucci, Sílvio Roberto Consonni, Daniel Martins-de-Souza, Marcelo Bispo de Jesus, Steven Thomas Russell and Maria Cristina Cintra Gomes-Mardondes

Gel_1		
CIV/Vinculin	Mean of C	Ratio
0.275680774	0.318214	86.63372
0.360747566		113.3663
0.330918326		103.9923
0.34548761		108.5708
0.117575475		36.94854
0.158820791		49.91003
0.449793063		141.3492
0.266146436		83.63752

Gel_1		
CI/Vinculin	Mean of C	Ratio
0.514979	0.637459	80.78615938
0.759939		119.2138406
1.02493		160.7836489
0.99849		156.6359941
0.571878		89.71214929
0.606096		95.07999954
0.766829		120.2945406
0.636414		99.83602837

Gel_2		
CIV/Vinculin	Mean of C	Ratio
0.428059967	0.619645	69.08153
0.811229202		130.9185
0.543169056		87.65816
0.393130918		63.44458
0.192651984		31.09072
0.40063051		64.65489
0.438857918		70.82414
0.306759908		49.50578

Gel_2		
CI/Vinculin	Mean of C	Ratio
	0.775722	
0.775722		100
1.040769		134.1678552
0.211823		27.30662199
0.456835		58.89165813
0.231621		29.85879315
0.195921		25.25662188
0.572322		73.7792588

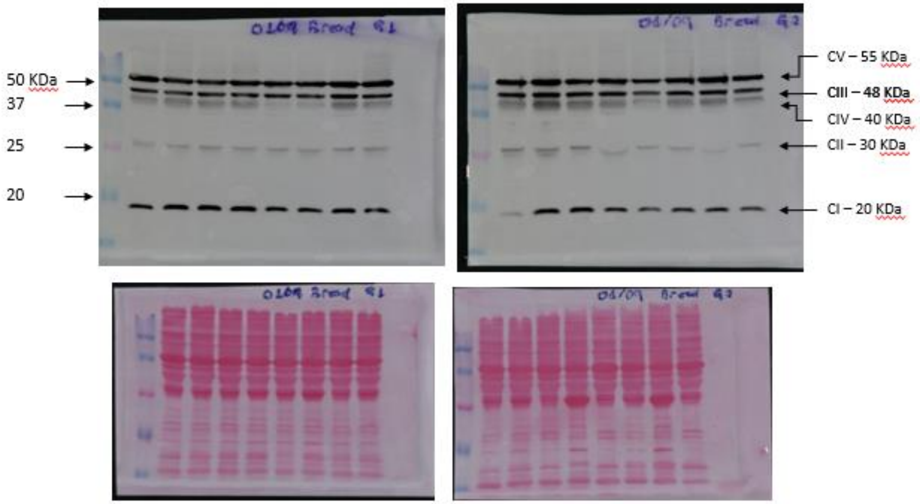

Gel_1		
p-Creb/Vinculin	Mean of C	Ratio
0.581117882	0.696496	83.43444
0.811874863		116.5656
0.705245453		101.2562
0.776825833		111.5334
1.908414527		274.0021
2.219475389		318.6629
0.907165878		130.247
0.448428394		64.38345

Gel_2		
p-Creb/Vinculin	Mean of C	Ratio
4.011419362		
1.544140536	1.544141	100
1.980137366		128.2356
1.313271975		85.04873
4.271650694		276.6361
2.866000498		185.6049
0.77762518		50.35974
0.900987286		58.34879

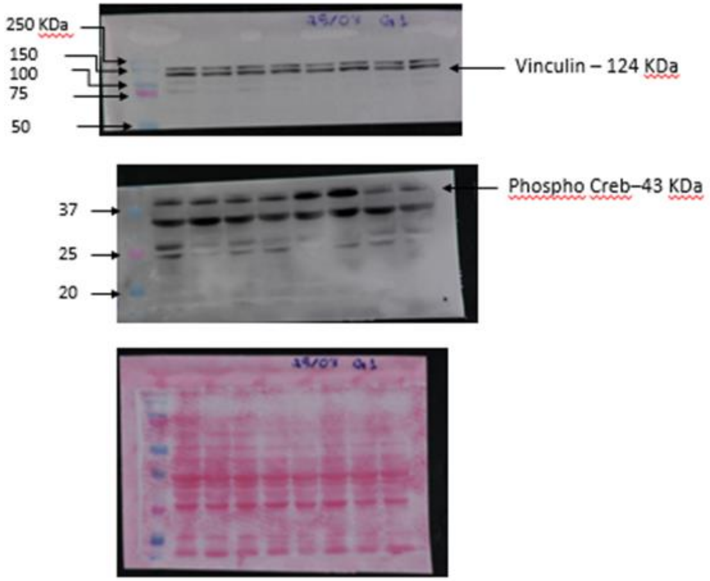

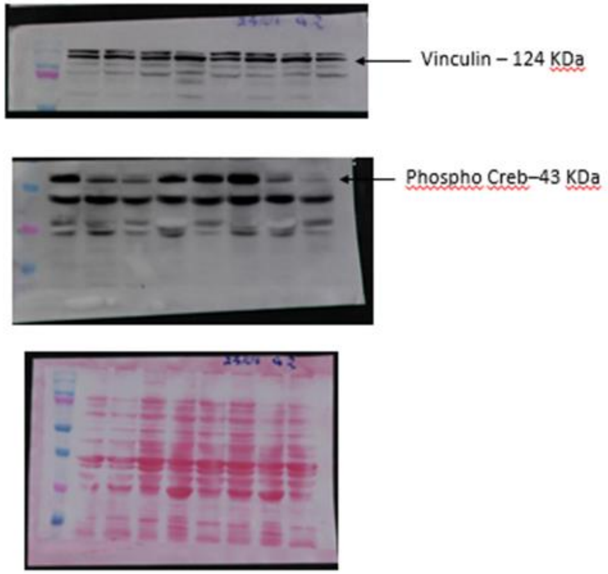

Figure S1. Detailed information about western blot in Figure 6.

Table S1. Total skeletal muscle metabolic profile identified in rats euthanatized at 7th day of the experiment.

Metabolite	C 7 Mean ± SD (mM)	L 7 Mean ± SD (mM)	W 7 Mean ± SD (mM)	WL 7 Mean ± SD (mM)	*p* value
2-Hydroxyisobutyrate	0.0307 ± 0.0157	0.0271 ± 0.0109	0.0196 ± 0.0056	0.0339 ± 0.0145	0.432
5,6-Dihydrothymine	0.3135 ± 0.1855	0.3072 ± 0.2264	0.3447 ± 0.1242	0.2894 ± 0.0772	0.970
ADP	0.3619 ± 0.2529	0.5366 ± 0.3415	0.1881 ± 0.1327	0.3090 ± 0.1025	0.240
AMP	0.0593 ± 0.0260	0.0462 ± 0.0193	0.0587 ± 0.0265	0.0628 ± 0.0309	0.817
ATP	0.0433 ± 0.0317	0.0304 ± 0.0103	0.0319 ± 0.0118	0.0388 ± 0.0164	0.765
Acetate	1.0418 ± 0.7943	0.2557 ± 0.1462	0.7260 ± 0.5012	0.5849 ± 0.2952	0.216
Alanine	4.0665 ± 2.1213	3.9517 ± 1.2937	3.7843 ± 1.0968	4.3584 ± 1.0101	0.952
Anserine	4.0762 ± 5.7168	0.0966 ± 0.0122	0.0955 ± 0.0681	0.1022 ± 0.0791	0.178
Ascorbate	1.1292 ± 0.7133	1.5219 ± 0.5562	1.6517 ± 0.4228	1.6328 ± 0.5660	0.560
Carnosine	3.3706 ± 1.9132	2.9143 ± 0.5788	2.4496 ± 1.6897	3.5031 ± 2.4611	0.836
Creatine	44.3915 ± 19.5185	30.4124 ± 5.5437	36.9566 ± 9.5283	34.8272 ± 9.6078	0.462
Creatine phosphate	0.4060 ± 0.1809	0.0904 ± 0.0836	0.4079 ± 0.2796	0.6346 ± 0.5495	0.182
Creatinine	0.5812 ± 0.2931	0.4002 ± 0.0759	0.6065 ± 0.1290	0.4207 ± 0.2669	0.420
Ethanol	0.1999 ± 0.1922	0.1603 ± 0.0927	0.0958 ± 0.0411	0.0914 ± 0.0360	0.466
Formate	0.3833 ± 0.2495	0.2202 ± 0.0569	0.4161 ± 0.1918	0.3781 ± 0.1277	0.410
Fumarate	0.1686 ± 0.0919	0.1763 ± 0.0880	0.1598 ± 0.0521	0.1588 ± 0.0251	0.982
Glucose	2.8980 ± 2.4123	1.5286 ± 0.5475	2.5253 ± 0.4909	2.1417 ± 0.7593	0.523
Glucose-1-phosphate	0.2911 ± 0.1491	0.1216 ± 0.0142	0.2489 ± 0.0820	0.2842 ± 0.1099	0.118
Glucose-6-phosphate	3.0885 ± 1.7516	0.8264 ± 0.1496	2.9016 ± 1.0911	3.2799 ± 1.7332	0.081
Glutamate	1.2576 ± 0.6533	2.0700 ± 0.9594	1.5909 ± 0.5283	1.6377 ± 0.7913	0.523
Glutamine	3.2098 ± 1.8013	3.7628 ± 3.1131	3.3612 ± 1.1906	4.2098 ± 1.8377	0.907
Glycerol	0.9306 ± 0.7770	1.5961 ± 0.8273	2.7112 ± 0.4269	1.5330 ± 0.3722	0.013
Glycine	3.3228 ± 2.0279	1.1678 ± 0.1664	2.2952 ± 0.6924	1.6309 ± 0.6017	0.085
IMP	4.7320 ± 2.2176	3.1616 ± 0.6800	4.5099 ± 0.9254	4.5916 ± 1.4109	0.411
Inosine	0.1618 ± 0.0821	0.1620 ± 0.1305	0.1372 ± 0.0291	0.1995 ± 0.0861	0.804
Lactate	64.2890 ± 31.6685	43.4124 ± 4.0930	57.0051 ± 14.3153	50.4049 ± 16.5041	0.491
Leucine	0.2314 ± 0.1297	0.3456 ± 0.0348	0.1758 ± 0.0518	0.3927 ± 0.0477	0.006
Lysine	0.8365 ± 0.5052	0.8012 ± 0.5443	0.6754 ± 0.2552	0.5683 ± 0.4182	0.822
Malonate	0.1085 ± 0.0670	0.0818 ± 0.0268	0.0843 ± 0.0556	0.1547 ± 0.0875	0.370
Methionine	0.1148 ± 0.0384	0.0938 ± 0.0234	0.0652 ± 0.0375	0.3812 ± 0.1771	0.001
NAD+	0.3261 ± 0.1806	0.2433 ± 0.0597	0.2079 ± 0.0523	0.1730 ± 0.0675	0.245

Niacinamide	0.3693 ± 0.1992	0.2871 ± 0.1149	0.4194 ± 0.1105	0.4489 ± 0.0667	0.368
O-Acetylcarnitine	0.6142 ± 0.3278	0.4876 ± 0.1737	0.4467 ± 0.2726	0.2858 ± 0.2527	0.400
Pyruvate	0.0890 ± 0.0535	0.0463 ± 0.0112	0.1286 ± 0.1470	0.0897 ± 0.0292	0.564
Sarcosine	0.0774 ± 0.0369	0.0834 ± 0.0144	0.0791 ± 0.0285	0.0848 ± 0.0185	0.974
Succinate	0.6369 ± 0.4278	0.8098 ± 0.4044	0.4344 ± 0.1393	0.4443 ± 0.3017	0.381
Taurine	28.0479 ± 13.0051	29.4458 ± 12.7660	28.1842 ± 8.8871	28.6319 ± 5.0354	0.997
Threonine	0.5777 ± 0.5406	0.4275 ± 0.0660	0.5565 ± 0.2623	0.5504 ± 0.4774	0.944
Tyrosine	0.2074 ± 0.0976	0.1731 ± 0.0261	0.1977 ± 0.0691	0.1964 ± 0.0273	0.884
Valine	0.3104 ± 0.1555	0.2724 ± 0.0697	0.2293 ± 0.0654	0.2113 ± 0.0326	0.460
β-Alanine	0.2016 ± 0.1044	0.1870 ± 0.0788	0.1388 ± 0.0260	0.1974 ± 0.0831	0.663
Methylhistidine	0.0375 ± 0.0208	0.0972 ± 0.0545	0.0969 ± 0.0523	0.0790 ± 0.0790	0.417

Rats were distributed into control (C7); fed a leucine-rich diet (L7); Walker tumour-bearing (W7) and Walker tumour-bearing fed a leucine-rich diet (WL7) euthanatized at 7th day of the experiment. Data were expressed as mean ± standard deviation (SD) and analysed by one-way ANOVA (comparison among C7, L7, W7 and WL7). Bold *p* values represented a significant difference.

Table S2. Total skeletal muscle metabolic profile identified in rats euthanatized at 14th day of the experiment.

**Metabolite**	**C 14**	**L 14**	**W 14**	**WL 14**	***p* Value**
	**Mean ± SD (mM)**	**Mean ± SD (mM)**	**Mean ± SD (mM)**	**Mean ± SD (mM)**	
2-Hydroxyisobutyrate	0.0307 ± 0.0157	0.0472 ± 0.0024	0.0262 ± 0.0195	0.0227 ± 0.0062	0.085
5,6-Dihydrothymine	0.3137 ± 0.1859	0.3320 ± 0.0144	0.4500 ± 0.0418	0.2813 ± 0.0424	0.134
ADP	0.3620 ± 0.2529	0.5429 ± 0.0333	0.3265 ± 0.1107	0.3626 ± 0.0679	0.189
AMP	0.0594 ± 0.0260	0.0638 ± 0.0110	0.0769 ± 0.0205	0.1004 ± 0.0629	0.407
ATP	0.0434 ± 0.0317	0.0463 ± 0.0116	0.0668 ± 0.0479	0.0377 ± 0.0091	0.551
Acetate	1.0423 ± 0.7945	0.3235 ± 0.1469	1.3057 ± 0.6831	0.8249 ± 0.3115	0.131
Alanine	4.0684 ± 2.1228	4.8933 ± 0.0625	4.3933 ± 0.2019	5.1546 ± 0.8497	0.557
Anserine	4.0806 ± 5.7268	0.0666 ± 0.0266	0.2080 ± 0.2463	0.1746 ± 0.1120	0.186
Ascorbate	1.1294 ± 0.7132	0.8371 ± 0.5842	1.7339 ± 0.3261	1.4115 ± 0.2142	0.124
Carnosine	3.3725 ± 1.9157	3.3095 ± 0.0018	4.8081 ± 0.6785	4.0164 ± 0.5616	0.209
Creatine	44.4119 ± 19.5488	37.4204 ± 3.2481	52.2857 ± 4.3232	41.0840 ± 1.4348	0.252
Creatine phosphate	0.4060 ± 0.1809	0.1811 ± 0.0260	0.3042 ± 0.2813	0.3250 ± 0.3551	0.639
Creatinine	0.5815 ± 0.2935	0.3702 ± 0.0135	0.7157 ± 0.0496	0.6153 ± 0.0885	0.050
Ethanol	0.2000 ± 0.1926	0.1275 ± 0.0460	0.1310 ± 0.0701	0.0847 ± 0.0901	0.571
Formate	0.3835 ± 0.2498	0.2895 ± 0.0204	0.6032 ± 0.1839	0.4575 ± 0.1420	0.119

Fumarate	0.1687 ± 0.0920	0.1693 ± 0.0030	0.1781 ± 0.0343	0.1850 ± 0.0449	0.968
Glucose	2.9004 ± 2.4169	1.4456 ± 0.4042	1.8538 ± 0.9147	2.1494 ± 0.4338	0.489
Glucose-1-phosphate	0.2913 ± 0.1494	0.1388 ± 0.0063	0.1739 ± 0.0867	0.2644 ± 0.0315	0.091
Glucose-6-phosphate	3.0903 ± 1.7551	0.8283 ± 0.1405	2.3611 ± 1.2505	2.9234 ± 0.1588	0.045
Glutamate	1.2582 ± 0.6540	1.4199 ± 0.0916	1.2259 ± 0.3348	1.8763 ± 0.7381	0.315
Glutamine	3.2109 ± 1.8015	2.8937 ± 0.7215	2.9475 ± 0.4088	4.0777 ± 1.7318	0.574
Glycerol	0.9307 ± 0.7770	0.5856 ± 0.0662	0.8393 ± 0.1139	0.9789 ± 0.1251	0.532
Glycine	3.3249 ± 2.0323	1.9920 ± 0.4937	2.8609 ± 0.7782	2.6599 ± 0.3377	0.442
IMP	4.7342 ± 2.2210	4.3544 ± 0.9823	5.0563 ± 1.0573	4.5896 ± 0.4451	0.900
Inosine	0.1618 ± 0.0822	0.1242 ± 0.0413	0.1344 ± 0.0317	0.1412 ± 0.0585	0.815
Lactate	64.3203 ± 31.7140	48.9957 ± 4.8405	56.4696 ± 15.4306	58.3740 ± 7.6167	0.701
Leucine	0.2315 ± 0.1297	0.3741 ± 0.0872	0.2730 ± 0.0640	0.2537 ± 0.0376	0.151
Lysine	0.8368 ± 0.5054	0.6026 ± 0.0321	1.0468 ± 0.1850	1.1623 ± 0.3119	0.109
Malonate	0.1085 ± 0.0671	0.1213 ± 0.0430	0.1834 ± 0.1049	0.0693 ± 0.0272	0.170
Methionine	0.1149 ± 0.0385	0.0816 ± 0.0070	0.1511 ± 0.2442	0.0708 ± 0.0320	0.799
NAD+	0.3262 ± 0.1807	0.3890 ± 0.0027	0.3826 ± 0.1050	0.2764 ± 0.0094	0.417
Niacinamide	0.3695 ± 0.1994	0.3197 ± 0.0269	0.4358 ± 0.0983	0.4016 ± 0.0670	0.562
O-Acetylcarnitine	0.6144 ± 0.3278	0.6521 ± 0.0268	0.4632 ± 0.3096	0.3073 ± 0.2766	0.287
Pyruvate	0.0890 ± 0.0536	0.0560 ± 0.0048	0.1210 ± 0.0587	0.0874 ± 0.0232	0.233
Sarcosine	0.0775 ± 0.0369	0.1084 ± 0.0029	0.1130 ± 0.0197	0.0867 ± 0.0215	0.155
Succinate	0.6371 ± 0.4278	0.7984 ± 0.0609	0.5683 ± 0.1306	0.5916 ± 0.1139	0.521
Taurine	28.0577 ± 13.0109	32.3942 ± 0.5020	31.6355 ± 3.6816	31.4140 ± 6.3537	0.849
Threonine	0.5778 ± 0.5406	0.4557 ± 0.1088	0.5248 ± 0.2253	0.7597 ± 0.3794	0.662
Tyrosine	0.2075 ± 0.0977	0.1779 ± 0.0130	0.2023 ± 0.0587	0.2047 ± 0.0201	0.881
Valine	0.3105 ± 0.1557	0.2315 ± 0.0021	0.2912 ± 0.0573	0.2440 ± 0.0444	0.534
β-Alanine	0.2017 ± 0.1045	0.1986 ± 0.0051	0.1666 ± 0.0331	0.1948 ± 0.0447	0.827
Methylhistidine	0.0375 ± 0.0207	0.1526 ± 0.0975	0.0780 ± 0.0749	0.1799 ± 0.1699	0.251

Rats were distributed into control (C14); fed a leucine-rich diet (L14); Walker tumour-bearing (W14) and Walker tumour-bearing fed a leucine-rich diet (WL14) euthanatized at 14th day of the experiment. Data were expressed as mean ± standard deviation (SD) and analysed by one-way ANOVA (comparison among C14, L14, W14 and WL14). Bold *p* values represented a significant difference.

Table S3. Total skeletal muscle metabolic profile identified in rats euthanatized at 21st day of the experiment.

Metabolite	C 21 Mean ± SD (mM)	L 21 Mean ± SD (mM)	W 21 Mean ± SD (mM)	WL 21 Mean ± SD (mM)	*p* Value
2-Hydroxyisobutyrate	0.0307 ± 0.0158	0.0248 ± 0.0091	0.0113 ± 0.0066	0.0178 ± 0.0059	0.087
5,6-Dihydrothymine	0.3133 ± 0.1866	0.1318 ± 0.0592	0.3473 ± 0.1006	0.3427 ± 0.0334	0.054
ADP	0.3615 ± 0.2532	0.2630 ± 0.2255	0.4218 ± 0.0606	0.2053 ± 0.1363	0.387
AMP	0.0592 ± 0.0260	0.0407 ± 0.0194	0.0630 ± 0.0110	0.0494 ± 0.0122	0.344
ATP	0.0433 ± 0.0317	0.0410 ± 0.0331	0.0439 ± 0.0127	0.0491 ± 0.0099	0.970
Acetate	1.0414 ± 0.7950	0.4141 ± 0.1759	0.9172 ± 0.2704	0.9328 ± 0.5644	0.354
Alanine	4.0614 ± 2.1222	3.5457 ± 1.1439	4.4503 ± 0.6268	5.1928 ± 0.4204	0.349
Anserine	4.0760 ± 5.7335	0.2169 ± 0.3337	0.1478 ± 0.1226	0.0497 ± 0.0220	0.186
Ascorbate	1.1266 ± 0.7105	1.0785 ± 0.4434	2.0452 ± 1.4722	1.7517 ± 0.3498	0.349
Carnosine	3.3680 ± 1.9179	2.4183 ± 0.6374	2.5533 ± 1.5661	0.0514 ± 0.0248	0.018
Creatine	44.3321 ± 19.5919	26.9760 ± 7.6313	43.3585 ± 10.1315	42.2676 ± 3.9035	0.179
Creatine phosphate	0.4047 ± 0.1797	0.2726 ± 0.2292	0.1618 ± 0.0837	0.4766 ± 0.5745	0.547
Creatinine	0.5804 ± 0.2936	0.3219 ± 0.0956	0.4602 ± 0.0693	0.3988 ± 0.2332	0.340
Ethanol	0.1998 ± 0.1930	0.0959 ± 0.0196	0.0905 ± 0.0191	0.0472 ± 0.0026	0.208
Formate	0.3831 ± 0.2501	0.2563 ± 0.0970	0.4190 ± 0.1039	0.4424 ± 0.1582	0.417
Fumarate	0.1683 ± 0.0918	0.1621 ± 0.0571	0.1803 ± 0.0194	0.1551 ± 0.0346	0.936
Glucose	2.8976 ± 2.4203	1.2716 ± 0.1866	1.6230 ± 0.7941	2.8789 ± 0.1351	0.211
Glucose-1-phosphate	0.2908 ± 0.1496	0.1546 ± 0.0621	0.1768 ± 0.0518	0.2923 ± 0.0944	0.133
Glucose-6-phosphate	3.0849 ± 1.7576	1.1551 ± 0.2559	1.8279 ± 0.6449	4.5525 ± 1.6350	0.011
Glutamate	1.2560 ± 0.6537	1.4570 ± 1.0640	1.6838 ± 0.3261	1.1609 ± 0.1099	0.681
Glutamine	3.2042 ± 1.7969	3.3890 ± 1.1662	4.0799 ± 1.5466	3.7360 ± 0.3206	0.798
Glycerol	0.9280 ± 0.7744	0.6848 ± 0.2835	0.8751 ± 0.1446	0.9638 ± 0.3008	0.816
Glycine	3.3202 ± 2.0368	1.4577 ± 0.5637	2.9912 ± 1.1063	2.7522 ± 0.5475	0.205
IMP	4.7242 ± 2.2211	4.0784 ± 1.7489	4.6693 ± 1.2234	5.4110 ± 0.4046	0.694
Inosine	0.1616 ± 0.0823	0.1301 ± 0.0557	0.1226 ± 0.0530	0.1641 ± 0.0307	0.668
Lactate	64.1950 ± 31.7147	41.5969 ± 9.2174	62.1153 ± 18.1809	67.7295 ± 6.1134	0.260
Leucine	0.2309 ± 0.1293	0.2575 ± 0.0533	0.2330 ± 0.0447	0.3079 ± 0.0337	0.468
Lysine	0.8355 ± 0.5046	0.5333 ± 0.3183	1.1009 ± 0.4029	1.8592 ± 1.0032	0.054
Malonate	0.1083 ± 0.0673	0.0836 ± 0.0341	0.0833 ± 0.0150	0.1483 ± 0.0539	0.220
Methionine	0.1146 ± 0.0387	0.0717 ± 0.0299	0.0925 ± 0.0751	0.3916 ± 0.2459	0.015
NAD+	0.3258 ± 0.1811	0.2460 ± 0.1025	0.2820 ± 0.0836	0.1642 ± 0.0291	0.273

Niacinamide	0.3687 ± 0.1989	0.3064 ± 0.0661	0.4460 ± 0.0230	0.4880 ± 0.0795	0.160
O-Acetylcarnitine	0.6133 ± 0.3279	0.3674 ± 0.1723	0.4027 ± 0.2377	0.4158 ± 0.2112	0.505
Pyruvate	0.0889 ± 0.0536	0.0701 ± 0.0382	0.0820 ± 0.0335	0.1289 ± 0.0349	0.251
Sarcosine	0.0773 ± 0.0369	0.0659 ± 0.0164	0.0805 ± 0.0350	0.0681 ± 0.0231	0.870
Succinate	0.6362 ± 0.4283	0.4190 ± 0.1544	0.4329 ± 0.1446	0.3348 ± 0.0968	0.386
Taurine	27.9987 ± 12.9964	24.3398 ± 9.6149	29.3401 ± 0.9863	22.7046 ± 4.3095	0.660
Threonine	0.5764 ± 0.5396	0.4417 ± 0.0953	0.7510 ± 0.3818	1.1395 ± 0.2408	0.078
Tyrosine	0.2071 ± 0.0978	0.1239 ± 0.0365	0.2212 ± 0.0735	0.1829 ± 0.0533	0.255
Valine	0.3100 ± 0.1558	0.1893 ± 0.0645	0.2853 ± 0.0386	0.2559 ± 0.0484	0.306
β-Alanine	0.2013 ± 0.1043	0.1403 ± 0.0386	0.1846 ± 0.0567	0.1571 ± 0.0246	0.549
Methylhistidine	0.0373 ± 0.0205	0.0767 ± 0.0484	0.0787 ± 0.0905	0.0982 ± 0.0787	0.622

Rats were distributed into control (C21); fed a leucine-rich diet (L21); Walker tumour-bearing (W21) and Walker tumour-bearing fed a leucine-rich diet (WL21) euthanatized at 21st day of the experiment. Data were expressed as mean ± standard deviation (SD) and analysed by one-way ANOVA (comparison among C21, L21, W21 and WL21). Bold *p* values represented a significant difference

Table S4. Total skeletal muscle metabolic profile identified in rats euthanatized at 7th, 14th and 21st days of the experiment.

**Metabolite**	**W 7**	**W 14**	**W 21**	***p* Value**	**WL 7**	**WL 14**	**WL 21**	***p* Value**
	**Mean ± SD (mM)**	**Mean ± SD (mM)**	**Mean ± SD (mM)**		**Mean ± SD (mM)**	**Mean ± SD (mM)**	**Mean ± SD (mM)**	
2-Hydroxyisobutyrate	0.0196 ± 0.0056	0.0262 ± 0.0195	0.0113 ± 0.0066	0.277	0.0339 ± 0.0145	0.0227 ± 0.0062	0.0178 ± 0.0059	0.109
5,6-Dihydrothymine	0.3447 ± 0.1242	0.4500 ± 0.0418	0.3473 ± 0.1006	0.257	0.2894 ± 0.0772	0.2813 ± 0.0424	0.3427 ± 0.0334	0.273
ADP	0.1881 ± 0.1327	0.3265 ± 0.1107	0.4218 ± 0.0606	0.036	0.3090 ± 0.1025	0.3626 ± 0.0679	0.2053 ± 0.1363	0.158
AMP	0.0587 ± 0.0265	0.0769 ± 0.0205	0.0630 ± 0.0110	0.448	0.0628 ± 0.0309	0.1004 ± 0.0629	0.0494 ± 0.0122	0.244
ATP	0.0319 ± 0.0118	0.0668 ± 0.0479	0.0439 ± 0.0127	0.283	0.0388 ± 0.0164	0.0377 ± 0.0091	0.0491 ± 0.0099	0.388
Acetate	0.7260 ± 0.5012	1.3057 ± 0.6831	0.9172 ± 0.2704	0.313	0.5849 ± 0.2952	0.8249 ± 0.3115	0.9328 ± 0.5644	0.497
Alanine	3.7843 ± 1.0968	4.3933 ± 0.2019	4.4503 ± 0.6268	0.406	4.3584 ± 1.0101	5.1546 ± 0.8497	5.1928 ± 0.4204	0.298
Anserine	0.0955 ± 0.0681	0.2080 ± 0.2463	0.1478 ± 0.1226	0.637	0.1022 ± 0.0791	0.1746 ± 0.1120	0.0497 ± 0.0220	0.142
Ascorbate	1.6517 ± 0.4228	1.7339 ± 0.3261	2.0452 ± 1.4722	0.814	1.6328 ± 0.5660	1.4115 ± 0.2142	1.7517 ± 0.3498	0.507
Carnosine	2.4496 ± 1.6897	4.8081 ± 0.6785	2.5533 ± 1.5661	0.067	3.5031 ± 2.4611	4.0164 ± 0.5616	0.0514 ± 0.0248	0.008
Creatine	36.9566 ± 9.5283	52.2857 ± 4.3232	43.3585 ± 10.1315	0.082	34.8272 ± 9.6078	41.0840 ± 1.4348	42.2676 ± 3.9035	0.228
Creatine phosphate	0.4079 ± 0.2796	0.3042 ± 0.2813	0.1618 ± 0.0837	0.369	0.6346 ± 0.5495	0.3250 ± 0.3551	0.4766 ± 0.5745	0.695
Creatinine	0.6065 ± 0.1290	0.7157 ± 0.0496	0.4602 ± 0.0693	0.009	0.4207 ± 0.2669	0.6153 ± 0.0885	0.3988 ± 0.2332	0.325
Ethanol	0.0958 ± 0.0411	0.1310 ± 0.0701	0.0905 ± 0.0191	0.465	0.0914 ± 0.0360	0.0847 ± 0.0901	0.0472 ± 0.0026	0.511
Formate	0.4161 ± 0.1918	0.6032 ± 0.1839	0.4190 ± 0.1039	0.238	0.3781 ± 0.1277	0.4575 ± 0.1420	0.4424 ± 0.1582	0.716
Fumarate	0.1598 ± 0.0521	0.1781 ± 0.0343	0.1803 ± 0.0194	0.711	0.1588 ± 0.0251	0.1850 ± 0.0449	0.1551 ± 0.0346	0.468
Glucose	2.5253 ± 0.4909	1.8538 ± 0.9147	1.6230 ± 0.7941	0.265	2.1417 ± 0.7593	2.1494 ± 0.4338	2.8789 ± 0.1351	0.117

Glucose-1-phosphate	0.2489 ± 0.0820	0.1739 ± 0.0867	0.1768 ± 0.0518	0.324	0.2842 ± 0.1099	0.2644 ± 0.0315	0.2923 ± 0.0944	0.895
Glucose-6-phosphate	2.9016 ± 1.0911	2.3611 ± 1.2505	1.8279 ± 0.6449	0.377	3.2799 ± 1.7332	2.9234 ± 0.1588	4.5525 ± 1.6350	0.265
Glutamate	1.5909 ± 0.5283	1.2259 ± 0.3348	1.6838 ± 0.3261	0.293	1.6377 ± 0.7913	1.8763 ± 0.7381	1.1609 ± 0.1099	0.308
Glutamine	3.3612 ± 1.1906	2.9475 ± 0.4088	4.0799 ± 1.5466	0.408	4.2098 ± 1.8377	4.0777 ± 1.7318	3.7360 ± 0.3206	0.896
Glycerol	2.7112 ± 0.4269	0.8393 ± 0.1139	0.8751 ± 0.1446	<0.001	1.5330 ± 0.3722	0.9789 ± 0.1251	0.9638 ± 0.3008	0.032
Glycine	2.2952 ± 0.6924	2.8609 ± 0.7782	2.9912 ± 1.1063	0.517	1.6309 ± 0.6017	2.6599 ± 0.3377	2.7522 ± 0.5475	0.022
IMP	4.5099 ± 0.9254	5.0563 ± 1.0573	4.6693 ± 1.2234	0.767	4.5916 ± 1.4109	4.5896 ± 0.4451	5.4110 ± 0.4046	0.361
Inosine	0.1372 ± 0.0291	0.1344 ± 0.0317	0.1226 ± 0.0530	0.859	0.1995 ± 0.0861	0.1412 ± 0.0585	0.1641 ± 0.0307	0.447
Lactate	57.0051 ± 14.3153	56.4696 ± 15.4306	62.1153 ± 18.1809	0.862	50.4049 ± 16.5041	58.3740 ± 7.6167	67.7295 ± 6.1134	0.141
Leucine	0.1758 ± 0.0518	0.2730 ± 0.0640	0.2330 ± 0.0447	0.086	0.3927 ± 0.0477	0.2537 ± 0.0376	0.3079 ± 0.0337	0.003
Lysine	0.6754 ± 0.2552	1.0468 ± 0.1850	1.1009 ± 0.4029	0.140	0.5683 ± 0.4182	1.1623 ± 0.3119	1.8592 ± 1.0032	0.060
Malonate	0.0843 ± 0.0556	0.1834 ± 0.1049	0.0833 ± 0.0150	0.115	0.1547 ± 0.0875	0.0693 ± 0.0272	0.1483 ± 0.0539	0.146
Methionine	0.0652 ± 0.0375	0.1511 ± 0.2442	0.0925 ± 0.0751	0.716	0.3812 ± 0.1771	0.0708 ± 0.0320	0.3916 ± 0.2459	0.049
NAD+	0.2079 ± 0.0523	0.3826 ± 0.1050	0.2820 ± 0.0836	0.045	0.1730 ± 0.0675	0.2764 ± 0.0094	0.1642 ± 0.0291	0.008
Niacinamide	0.4194 ± 0.1105	0.4358 ± 0.0983	0.4460 ± 0.0230	0.909	0.4489 ± 0.0667	0.4016 ± 0.0670	0.4880 ± 0.0795	0.280
O-Acetylcarnitine	0.4467 ± 0.2726	0.4632 ± 0.3096	0.4027 ± 0.2377	0.950	0.2858 ± 0.2527	0.3073 ± 0.2766	0.4158 ± 0.2112	0.738
Pyruvate	0.1286 ± 0.1470	0.1210 ± 0.0587	0.0820 ± 0.0335	0.757	0.0897 ± 0.0292	0.0874 ± 0.0232	0.1289 ± 0.0349	0.137
Sarcosine	0.0791 ± 0.0285	0.1130 ± 0.0197	0.0805 ± 0.0350	0.218	0.0848 ± 0.0185	0.0867 ± 0.0215	0.0681 ± 0.0231	0.425
Succinate	0.4344 ± 0.1393	0.5683 ± 0.1306	0.4329 ± 0.1446	0.328	0.4443 ± 0.3017	0.5916 ± 0.1139	0.3348 ± 0.0968	0.227
Taurine	28.1842 ± 8.8871	31.6355 ± 3.6816	29.3401 ± 0.9863	0.684	28.6319 ± 5.0354	31.4140 ± 6.3537	22.7046 ± 4.3095	0.112
Threonine	0.5565 ± 0.2623	0.5248 ± 0.2253	0.7510 ± 0.3818	0.532	0.5504 ± 0.4774	0.7597 ± 0.3794	1.1395 ± 0.2408	0.138
Tyrosine	0.1977 ± 0.0691	0.2023 ± 0.0587	0.2212 ± 0.0735	0.874	0.1964 ± 0.0273	0.2047 ± 0.0201	0.1829 ± 0.0533	0.706
Valine	0.2293 ± 0.0654	0.2912 ± 0.0573	0.2853 ± 0.0386	0.264	0.2113 ± 0.0326	0.2440 ± 0.0444	0.2559 ± 0.0484	0.347
β-Alanine	0.1388 ± 0.0260	0.1666 ± 0.0331	0.1846 ± 0.0567	0.324	0.1974 ± 0.0831	0.1948 ± 0.0447	0.1571 ± 0.0246	0.550
Methylhistidine	0.0969 ± 0.0523	0.0780 ± 0.0749	0.0787 ± 0.0905	0.921	0.0790 ± 0.0790	0.1799 ± 0.1699	0.0982 ± 0.0787	0.465

Rats were distributed into Walker tumour-bearing (W7, W14 and W21) and Walker tumour-bearing fed a leucine-rich diet (WL7, WL14 and WL21) euthanized at 7th, 14th and 21st days of the experiment. Data were expressed as mean ± standard deviation (SD) and analysed by one-way ANOVA (comparison among W7, W14 and W21 and among WL7, WL14 and WL21). Bold *p* values represented a significant difference.

Table S5. Total myotube metabolic profile identified in C_2C_{12} cells.

Metabolite	C	L	WF	WFL	*p* Value
	Mean ± SD (mM)	Mean ± SD (mM)	Mean ± SD (mM)	Mean ± SD (mM)	
2'-Deoxyadenosine	0.0201 ± 0.0099	0.0178 ± 0.0084	0.0184 ± 0.0134	0.0305 ± 0.0149	0.469
4-Hydroxybenzoate	0.0025 ± 0.0013	0.0020 ± 0.0002	0.0026 ± 0.0013	0.0031 ± 0.0023	0.846
5-Hydroxylysine	0.0048 ± 0.0012	0.0047 ± 0.0013	0.0023 ± 0.0027	0.0040 ± 0.0016	0.245
Acetate	0.0579 ± 0.0369	0.0494 ± 0.0232	0.0377 ± 0.0254	0.0865 ± 0.0293	0.182
Adenine	0.0028 ± 0.0017	0.0020 ± 0.0003	0.0021 ± 0.0006	0.0017 ± 0.0000	0.456
ADP	0.0070 ± 0.0036	0.0092 ± 0.0055	0.0034 ± 0.0013	0.0103 ± 0.0071	0.253
Alanine	0.0104 ± 0.0057	0.0090 ± 0.0042	0.0071 ± 0.0042	0.0159 ± 0.0076	0.214
Alloisoleucine	0.0043 ± 0.0006	0.0034 ± 0.0014	0.0028 ± 0.0023	0.0057 ± 0.0028	0.259
Arginine	0.0012 ± 0.0006	0.0017 ± 0.0000	0.0017 ± 0.0000	0.0011 ± 0.0007	0.221
Aspartate	0.0053 ± 0.0026	0.0051 ± 0.0022	0.0024 ± 0.0012	0.0072 ± 0.0023	0.054
Choline	0.0015 ± 0.0007	0.0013 ± 0.0004	0.0009 ± 0.0013	0.0033 ± 0.0006	0.007
Creatine	0.0079 ± 0.0027	0.0075 ± 0.0033	0.0065 ± 0.0054	0.0139 ± 0.0108	0.432
Creatine phosphate	0.0039 ± 0.0012	0.0025 ± 0.0008	0.0025 ± 0.0020	0.0056 ± 0.0043	0.351
Dimethylamine	0.0007 ± 0.0005	0.0007 ± 0.0003	0.0005 ± 0.0001	0.0014 ± 0.0002	0.005
Ethanol	0.0021 ± 0.0003	0.0400 ± 0.0548	0.0184 ± 0.0094	0.0089 ± 0.0097	0.261
Ethylene glycol	0.0012 ± 0.0010	0.0006 ± 0.0001	0.0015 ± 0.0009	0.0007 ± 0.0000	0.368
Formate	0.0140 ± 0.0065	0.0091 ± 0.0045	0.0093 ± 0.0065	0.0184 ± 0.0070	0.203
Glucose-6-phosphate	0.0117 ± 0.0023	0.0147 ± 0.0016	0.0082 ± 0.0031	0.0128 ± 0.0000	0.011
Glutamate	0.0404 ± 0.0139	0.0338 ± 0.0153	0.0340 ± 0.0200	0.0630 ± 0.0289	0.224
Glutamine	0.0146 ± 0.0054	0.0144 ± 0.0083	0.0102 ± 0.0070	0.0319 ± 0.0055	0.003
Glycine	0.0583 ± 0.0333	0.0512 ± 0.0229	0.0372 ± 0.0243	0.0711 ± 0.0349	0.473
Lactate	0.0558 ± 0.0293	0.0464 ± 0.0200	0.0406 ± 0.0335	0.0945 ± 0.0497	0.208
myo-Inositol	0.0198 ± 0.0108	0.0150 ± 0.0038	0.0160 ± 0.0118	0.0302 ± 0.0214	0.458
NAD+	0.0082 ± 0.0046	0.0066 ± 0.0019	0.0061 ± 0.0036	0.0121 ± 0.0056	0.268
N-Acetyllysine	0.0034 ± 0.0012	0.0055 ± 0.0001	0.0032 ± 0.0010	0.0105 ± 0.0013	<0.001
Proline	0.0069 ± 0.0029	0.0077 ± 0.0024	0.0044 ± 0.0037	0.0117 ± 0.0105	0.438
Pyruvate	0.0090 ± 0.0035	0.0083 ± 0.0030	0.0072 ± 0.0038	0.0127 ± 0.0051	0.286
Ribose	0.0042 ± 0.0017	0.0034 ± 0.0028	0.0025 ± 0.0029	0.0105 ± 0.0117	0.340
Sarcosine	0.0007 ± 0.0003	0.0008 ± 0.0002	0.0007 ± 0.0005	0.0019 ± 0.0002	0.001
sn-Glycero-3-phosphocholine	0.0036 ± 0.0020	0.0035 ± 0.0006	0.0042 ± 0.0026	0.0057 ± 0.0036	0.606
Taurine	0.0066 ± 0.0037	0.0052 ± 0.0034	0.0030 ± 0.0027	0.0077 ± 0.0024	0.223
Valerate	0.0053 ± 0.0023	0.0062 ± 0.0005	0.0059 ± 0.0017	0.0052 ± 0.0024	0.873

C_2C_{12} myotube cells were distributed into control (C); leucine supplementation medium (L); Walker Factor treatment (WF) and Walker Factor treatment and leucine supplementation medium (WFL). Data were expressed as mean ± standard deviation (SD) and analysed by one-way ANOVA (comparison among C, L, WF and WFL). Bold *p* values represented a significant difference.

Table S6. Comparison of the highest and lowest proteins concentration identified in muscle of rats from L and WL groups.

Accession	Description	Peptide Count	Unique Peptides	Confidence Score	Max Fold Change	Highest Mean Condition	Lowest Mean Condition
P62260; P35213; P61983; P63102; P68255; P68511	14-3-3 protein epsilon OS = Rattus norvegicus GN = Ywhae PE = 1 SV = 1	4	4	36,458	1355	WL	L
Q5XI78	2-oxoglutarate dehydrogenase, mitochondrial OS = Rattus norvegicus GN = Ogdh PE = 1 SV = 1	10	9	70,747	3326	L	WL
P13437	3-ketoacyl-CoA thiolase, mitochondrial OS = Rattus norvegicus GN = Acaa2 PE = 2 SV = 1	2	2	18,358	>5	L	WL
P06761	78 kDa glucose-regulated protein OS = Rattus norvegicus GN = Hspa5 PE = 1 SV = 1	6	5	39,225	1163	L	WL
P68136	Actin, alpha skeletal muscle OS = Rattus norvegicus GN = Acta1 PE = 1 SV = 1	4	4	40,114	2469	WL	L
P39069	Adenylate kinase isoenzyme 1 OS = Rattus norvegicus GN = Ak1 PE = 1 SV = 3	5	5	60,101	3043	WL	L
P23928	Alpha-crystallin B chain OS = Rattus norvegicus GN = Cryab PE = 1 SV = 1	3	3	28,582	3245	L	WL
P04764	Alpha-enolase OS = Rattus norvegicus GN = Eno1 PE = 1 SV = 4	7	4	73,461	2845	L	WL
P48037	Annexin A6 OS = Rattus norvegicus GN = Anxa6 PE = 1 SV = 2	11	11	78,329	>5	L	WL
P00507	Aspartate aminotransferase, mitochondrial OS = Rattus norvegicus GN = Got2 PE = 1 SV = 2	8	7	74,085	1825	WL	L
P19511	ATP synthase F(0) complex subunit B1, mitochondrial OS = Rattus norvegicus GN = Atp5f1 PE = 1 SV = 1	5	4	43,226	4772	L	WL
P15999	ATP synthase subunit alpha, mitochondrial OS = Rattus norvegicus GN = Atp5a1 PE = 1 SV = 2	12	12	131,546	2633	L	WL
P35435	ATP synthase subunit gamma, mitochondrial OS = Rattus norvegicus GN = Atp5c1 PE = 1 SV = 2	2	2	18,563	1924	L	WL
P15429	Beta-enolase OS = Rattus norvegicus GN = Eno3 PE = 1 SV = 3	10	7	157,566	>5	L	WL
P14141	Carbonic anhydrase 3 OS = Rattus norvegicus GN = Ca3 PE = 1 SV = 3	10	10	135,047	1292	L	WL
Q8VHF5	Citrate synthase, mitochondrial OS = Rattus norvegicus GN = Cs PE = 1 SV = 1	7	7	53,481	1237	WL	L
P00564	Creatine kinase M-type OS = Rattus norvegicus GN = Ckm PE = 1 SV = 2	17	17	211,203	1169	WL	L
P09605	Creatine kinase S-type, mitochondrial OS = Rattus norvegicus GN = Ckmt2 PE = 1 SV = 2	6	5	61,595	1835	L	WL
Q5XIQ3	CXXC-type zinc finger protein 5 OS = Rattus norvegicus GN = Cxxc5 PE = 1 SV = 1	2	2	20,814	1892	WL	L

P46892	Cyclin-dependent kinase 11B OS = Rattus norvegicus GN = Cdk11b PE = 1 SV = 1	4	4	26,886	1703	WL	L
P32551	Cytochrome b-c1 complex subunit 2, mitochondrial OS = Rattus norvegicus GN = Uqcrc2 PE = 1 SV = 2	5	5	43,010	2638	L	WL
P00406	Cytochrome c oxidase subunit 2 OS = Rattus norvegicus GN = Mtco2 PE = 1 SV = 3	3	2	25,674	1553	L	WL
P10888	Cytochrome c oxidase subunit 4 isoform 1, mitochondrial OS = Rattus norvegicus GN = Cox4i1 PE = 1 SV = 1	2	2	20,864	>5	L	WL
P11240	Cytochrome c oxidase subunit 5A, mitochondrial OS = Rattus norvegicus GN = Cox5a PE = 1 SV = 1	3	3	21,886	1468	L	WL
P62898	Cytochrome c, somatic OS = Rattus norvegicus GN = Cycs PE = 1 SV = 2	4	4	30,048	1371	L	WL
P08461	Dihydrolipoyllysine-residue acetyltransferase component of pyruvate dehydrogenase complex, mitochondrial OS = Rattus norvegicus GN = Dlat PE = 1 SV = 3	4	3	19,285	1444	L	WL
P13803	Electron transfer flavoprotein subunit alpha, mitochondrial OS = Rattus norvegicus GN = Etfa PE = 1 SV = 4	9	9	61,003	1843	L	WL
P62632; P62630	Elongation factor 1-alpha 2 OS = Rattus norvegicus GN = Eef1a2 PE = 1 SV = 1	6	6	54,214	1156	L	WL
P07483	Fatty acid-binding protein, heart OS = Rattus norvegicus GN = Fabp3 PE = 1 SV = 2	8	8	61,154	1686	L	WL
Q9Z1N1	Fructose-1,6-bisphosphatase isozyme 2 OS = Rattus norvegicus GN = Fbp2 PE = 1 SV = 1	3	3	42,543	1877	L	WL
P09117	Fructose-bisphosphate aldolase C OS = Rattus norvegicus GN = Aldoc PE = 1 SV = 3	4	2	25,730	>5	L	WL
Q6P6V0	Glucose-6-phosphate isomerase OS = Rattus norvegicus GN = Gpi PE = 1 SV = 1	15	15	131,781	1170	WL	L
P08010; P04905; P08009	Glutathione S-transferase Mu 2 OS = Rattus norvegicus GN = Gstm2 PE = 1 SV = 2	4	4	38,611	2061	WL	L
P04797; Q9ESV6	Glyceraldehyde-3-phosphate dehydrogenase OS = Rattus norvegicus GN = Gapdh PE = 1 SV = 3	28	28	363,412	1160	WL	L
O35077	Glycerol-3-phosphate dehydrogenase [NAD(+)], cytoplasmic OS = Rattus norvegicus GN = Gpd1 PE = 1 SV = 4	6	6	48,979	3049	L	WL
P09812	Glycogen phosphorylase, muscle form OS = Rattus norvegicus GN = Pygm PE = 1 SV = 5	24	19	323,311	1643	WL	L
O08730	Glycogenin-1 OS = Rattus norvegicus GN = Gyg1 PE = 2 SV = 4	2	2	10,252	1222	L	WL
P63018	Heat shock cognate 71 kDa protein OS = Rattus norvegicus GN = Hspa8 PE = 1 SV = 1	11	9	130,074	1608	L	WL
P42930	Heat shock protein beta-1 OS = Rattus norvegicus GN = Hspb1 PE = 1 SV = 1	3	3	19,092	3991	L	WL
P97541	Heat shock protein beta-6 OS = Rattus norvegicus GN = Hspb6 PE = 1 SV = 1	3	3	17,277	2251	L	WL

P34058	Heat shock protein HSP 90-beta OS = Rattus norvegicus GN = Hsp90ab1 PE = 1 SV = 4	5	5	34,725	>5	L	WL
P14659	Heat shock-related 70 kDa protein 2 OS = Rattus norvegicus GN = Hspa2 PE = 1 SV = 2	6	3	55,017	3637	L	WL
P01946	Hemoglobin subunit alpha-1/2 OS = Rattus norvegicus GN = Hba1 PE = 1 SV = 3	4	4	60,014	1710	L	WL
Q9WVK7	Hydroxyacyl-coenzyme A dehydrogenase, mitochondrial OS = Rattus norvegicus GN = Hadh PE = 2 SV = 1	4	4	25,213	1154	WL	L
Q99NA5	Isocitrate dehydrogenase [NAD] subunit alpha, mitochondrial OS = Rattus norvegicus GN = Idh3a PE = 1 SV = 1	3	3	34,410	3265	L	WL
P56574	Isocitrate dehydrogenase [NADP], mitochondrial OS = Rattus norvegicus GN = Idh2 PE = 1 SV = 2	8	8	62,297	1621	L	WL
P04642	L-lactate dehydrogenase A chain OS = Rattus norvegicus GN = Ldha PE = 1 SV = 1	21	20	224,752	3930	L	WL
O88989	Malate dehydrogenase, cytoplasmic OS = Rattus norvegicus GN = Mdh1 PE = 1 SV = 3	8	8	90,321	>5	L	WL
P04636	Malate dehydrogenase, mitochondrial OS = Rattus norvegicus GN = Mdh2 PE = 1 SV = 2	8	8	100,903	2614	WL	L
P08503	Medium-chain specific acyl-CoA dehydrogenase, mitochondrial OS = Rattus norvegicus GN = Acadm PE = 1 SV = 1	4	4	27,934	1128	L	WL
B0BMX9	Meiosis-specific with OB domain-containing protein OS = Rattus norvegicus GN = Meiob PE = 2 SV = 1	3	3	14,247	1783	L	WL
O08839	Myc box-dependent-interacting protein 1 OS = Rattus norvegicus GN = Bin1 PE = 1 SV = 1	5	5	26,946	>5	WL	L
P02600	Myosin light chain 1/3, skeletal muscle isoform OS = Rattus norvegicus GN = Myl1 PE = 1 SV = 2	5	4	42,748	>5	WL	L
P08733	Myosin regulatory light chain 2, ventricular/cardiac muscle isoform OS = Rattus norvegicus GN = Myl2 PE = 1 SV = 2	3	3	23,679	2696	L	WL
Q66HF1	NADH-ubiquinone oxidoreductase 75 kDa subunit, mitochondrial OS = Rattus norvegicus GN = Ndufs1 PE = 1 SV = 1	14	14	98,791	1393	WL	L
P02625	Parvalbumin alpha OS = Rattus norvegicus GN = Pvalb PE = 1 SV = 2	9	9	109,286	1462	WL	L
Q63716	Peroxiredoxin-1 OS = Rattus norvegicus GN = Prdx1 PE = 1 SV = 1	4	4	31,655	4880	L	WL
P31044	Phosphatidylethanolamine-binding protein 1 OS = Rattus norvegicus GN = Pebp1 PE = 1 SV = 3	3	3	46,882	>5	L	WL
P38652	Phosphoglucomutase-1 OS = Rattus norvegicus GN = Pgm1 PE = 1 SV = 2	11	11	127,683	3140	L	WL
P16617	Phosphoglycerate kinase 1 OS = Rattus norvegicus GN = Pgk1 PE = 1 SV = 2	10	10	103,270	1851	L	WL
P16290	Phosphoglycerate mutase 2 OS = Rattus norvegicus GN = Pgam2 PE = 1 SV = 2	7	5	94,841	1146	L	WL
P85125	Polymerase I and transcript release factor OS = Rattus norvegicus GN = Ptrf PE = 1 SV = 1	2	2	10,476	2708	WL	L
O88767	Protein DJ-1 OS = Rattus norvegicus GN = Park7 PE = 1 SV = 1	3	3	35,802	2144	WL	L
Q66HG8	Protein Red OS = Rattus norvegicus GN = Ik PE = 1 SV = 1	5	5	30,156	1832	WL	L

P49432	Pyruvate dehydrogenase E1 component subunit beta, mitochondrial OS = Rattus norvegicus GN = Pdhb PE = 1 SV = 2	7	7	60,044	1175	L	WL
P11980	Pyruvate kinase PKM OS = Rattus norvegicus GN = Pkm PE = 1 SV = 3	16	16	188,098	4401	L	WL
Q64578	Sarcoplasmic/endoplasmic reticulum calcium ATPase 1 OS = Rattus norvegicus GN = Atp2a1 PE = 1 SV = 1	22	15	238,396	1164	WL	L
P11507	Sarcoplasmic/endoplasmic reticulum calcium ATPase 2 OS = Rattus norvegicus GN = Atp2a2 PE = 1 SV = 1	15	8	137,588	2739	L	WL
P02770	Serum albumin OS = Rattus norvegicus GN = Alb PE = 1 SV = 2	8	8	75,582	>5	L	WL
Q920L2	Succinate dehydrogenase [ubiquinone] flavoprotein subunit, mitochondrial OS = Rattus norvegicus GN = Sdha PE = 1 SV = 1	4	4	33,199	1459	L	WL
Q68VK5	Tetraspanin-5 OS = Rattus norvegicus GN = Tspan5 PE = 2 SV = 2	3	3	16,102	1544	WL	L
Q60587	Trifunctional enzyme subunit beta, mitochondrial OS = Rattus norvegicus GN = Hadhb PE = 1 SV = 1	4	4	22,741	1150	L	WL
P48500	Triosephosphate isomerase OS = Rattus norvegicus GN = Tpi1 PE = 1 SV = 2	7	7	87,426	1619	L	WL
Q6AY56	Tubulin alpha-8 chain OS = Rattus norvegicus GN = Tuba8 PE = 2 SV = 1	2	2	10,657	2408	L	WL
P45953	Very long-chain specific acyl-CoA dehydrogenase, mitochondrial OS = Rattus norvegicus GN = Acadvl PE = 1 SV = 1	6	6	32,084	3227	WL	L
Q9Z2L0	Voltage-dependent anion-selective channel protein 1 OS = Rattus norvegicus GN = Vdac1 PE = 1 SV = 4	8	8	109,869	1992	WL	L
P81155	Voltage-dependent anion-selective channel protein 2 OS = Rattus norvegicus GN = Vdac2 PE = 1 SV = 2	4	3	42,303	1158	L	WL
Q9R1Z0	Voltage-dependent anion-selective channel protein 3 OS = Rattus norvegicus GN = Vdac3 PE = 1 SV = 2	5	4	54,983	1206	L	WL
P54283	Voltage-dependent L-type calcium channel subunit beta-1 OS = Rattus norvegicus GN = Cacnb1 PE = 1 SV = 1	4	4	32,222	1340	L	WL

Rats were distributed into fed a leucine-rich diet (L) and Walker tumour-bearing fed a leucine-rich diet (WL) euthanazied at 21st day of the experiment. Statistical analysis described in Methods, showing the significant value for $p < 0.05$.

Table S7. Comparison of the highest and lowest proteins concentration identified in muscle of rats from W and WL groups.

Accession	Description	Peptide Count	Unique Peptides	Confidence Score	Max Fold Change	Highest Mean Condition	Lowest Mean Condition
Q5XI78	2-oxoglutarate dehydrogenase, mitochondrial OS = Rattus norvegicus GN = Ogdh PE = 1 SV = 1	17	17	139,372	1806	WL	W
P13437	3-ketoacyl-CoA thiolase, mitochondrial OS = Rattus norvegicus GN = Acaa2 PE = 2 SV = 1	4	4	24,121	2441	W	WL
P85968	6-phosphogluconate dehydrogenase, decarboxylating OS = Rattus norvegicus GN = Pgd PE = 1 SV = 1	2	2	12,325	>5	W	WL
P17764	Acetyl-CoA acetyltransferase, mitochondrial OS = Rattus norvegicus GN = Acat1 PE = 1 SV = 1	3	3	16,777	>5	WL	W
Q9ER34	Aconitate hydratase, mitochondrial OS = Rattus norvegicus GN = Aco2 PE = 1 SV = 2	11	11	102,772	1812	WL	W
P39069	Adenylate kinase isoenzyme 1 OS = Rattus norvegicus GN = Ak1 PE = 1 SV = 3	6	6	56,364	4506	WL	W
Q05962	ADP/ATP translocase 1 OS = Rattus norvegicus GN = Slc25a4 PE = 1 SV = 3	13	3	113,430	1552	W	WL
Q09073	ADP/ATP translocase 2 OS = Rattus norvegicus GN = Slc25a5 PE = 1 SV = 3	11	3	98,468	4144	WL	W
P23928	Alpha-crystallin B chain OS = Rattus norvegicus GN = Cryab PE = 1 SV = 1	5	5	42,204	1556	WL	W
P04764	Alpha-enolase OS = Rattus norvegicus GN = Eno1 PE = 1 SV = 4	7	3	81,889	2827	WL	W
P00762	Anionic trypsin-1 OS = Rattus norvegicus GN = Prss1 PE = 1 SV = 1	2	2	25,029	>5	W	WL
Q07936	Annexin A2 OS = Rattus norvegicus GN = Anxa2 PE = 1 SV = 2	2	2	17,852	>5	WL	W
P48037	Annexin A6 OS = Rattus norvegicus GN = Anxa6 PE = 1 SV = 2	8	8	59,785	4159	WL	W
P00507	Aspartate aminotransferase, mitochondrial OS = Rattus norvegicus GN = Got2 PE = 1 SV = 2	7	7	62,773	1197	WL	W
P19511	ATP synthase F(0) complex subunit B1, mitochondrial OS = Rattus norvegicus GN = Atp5f1 PE = 1 SV = 1	5	5	57,303	>5	WL	W
P15999	ATP synthase subunit alpha, mitochondrial OS = Rattus norvegicus GN = Atp5a1 PE = 1 SV = 2	15	15	164,465	1473	W	WL
P10719	ATP synthase subunit beta, mitochondrial OS = Rattus norvegicus GN = Atp5b PE = 1 SV = 2	27	27	374,743	4379	WL	W
P31399	ATP synthase subunit d, mitochondrial OS = Rattus norvegicus GN = Atp5h PE = 1 SV = 3	4	3	32,690	3049	WL	W
P29419	ATP synthase subunit e, mitochondrial OS = Rattus norvegicus GN = Atp5i PE = 1 SV = 3	3	3	27,891	1478	WL	W

P35435	ATP synthase subunit gamma, mitochondrial OS = Rattus norvegicus GN = Atp5c1 PE = 1 SV = 2	3	3	18,359	4359	WL	W
Q06647	ATP synthase subunit O, mitochondrial OS = Rattus norvegicus GN = Atp5o PE = 1 SV = 1	5	4	36,238	1421	WL	W
P47858; P30835	ATP-dependent 6-phosphofructokinase, muscle type OS = Rattus norvegicus GN = Pfkm PE = 1 SV = 3	16	16	166,094	1437	W	WL
P15429; P07323	Beta-enolase OS = Rattus norvegicus GN = Eno3 PE = 1 SV = 3	16	10	214,847	3420	WL	W
P19633	Calsequestrin-1 OS = Rattus norvegicus GN = Casq1 PE = 1 SV = 2	9	9	83,435	2819	W	WL
P14141	Carbonic anhydrase 3 OS = Rattus norvegicus GN = Ca3 PE = 1 SV = 3	9	9	130,252	4184	W	WL
Q8VHF5	Citrate synthase, mitochondrial OS = Rattus norvegicus GN = Cs PE = 1 SV = 1	9	9	78,561	3133	WL	W
P00564	Creatine kinase M-type OS = Rattus norvegicus GN = Ckm PE = 1 SV = 2	23	22	293,461	2016	W	WL
P09605; P25809	Creatine kinase S-type, mitochondrial OS = Rattus norvegicus GN = Ckmt2 PE = 1 SV = 2	10	10	101,219	4192	WL	W
P32551	Cytochrome b-c1 complex subunit 2, mitochondrial OS = Rattus norvegicus GN = Uqcrc2 PE = 1 SV = 2	6	6	52,733	1854	WL	W
P00406	Cytochrome c oxidase subunit 2 OS = Rattus norvegicus GN = Mtco2 PE = 1 SV = 3	3	3	33,075	1807	WL	W
P10888	Cytochrome c oxidase subunit 4 isoform 1, mitochondrial OS = Rattus norvegicus GN = Cox4i1 PE = 1 SV = 1	4	4	38,279	3776	WL	W
P11240	Cytochrome c oxidase subunit 5A, mitochondrial OS = Rattus norvegicus GN = Cox5a PE = 1 SV = 1	3	3	28,557	2577	WL	W
P12075	Cytochrome c oxidase subunit 5B, mitochondrial OS = Rattus norvegicus GN = Cox5b PE = 1 SV = 2	2	2	21,426	>5	WL	W
P62898	Cytochrome c, somatic OS = Rattus norvegicus GN = Cycs PE = 1 SV = 2	3	3	22,791	>5	WL	W
Q01205	Dihydrolipoyllysine-residue succinyltransferase component of 2-oxoglutarate dehydrogenase complex, mitochondrial OS = Rattus norvegicus GN = Dlst PE = 1 SV = 2	5	3	50,304	>5	WL	W
P13803	Electron transfer flavoprotein subunit alpha, mitochondrial OS = Rattus norvegicus GN = Etfa PE = 1 SV = 4	4	4	32,187	1344	WL	W
P62632	Elongation factor 1-alpha 2 OS = Rattus norvegicus GN = Eef1a2 PE = 1 SV = 1	5	5	52,986	1764	WL	W
P52844	Estrogen sulfotransferase, isoform 1 OS = Rattus norvegicus GN = Sult1e1 PE = 2 SV = 1	2	2	10,103	1251	W	WL
P07483	Fatty acid-binding protein, heart OS = Rattus norvegicus GN = Fabp3 PE = 1 SV = 2	4	4	49,737	1402	WL	W

P05065	Fructose-bisphosphate aldolase A OS = Rattus norvegicus GN = Aldoa PE = 1 SV = 2	15	15	196,230	1344	WL	W
P14408	Fumarate hydratase, mitochondrial OS = Rattus norvegicus GN = Fh PE = 1 SV = 1	5	5	41,588	>5	WL	W
Q6P6V0	Glucose-6-phosphate isomerase OS = Rattus norvegicus GN = Gpi PE = 1 SV = 1	17	17	152,797	4727	WL	W
P10860	Glutamate dehydrogenase 1, mitochondrial OS = Rattus norvegicus GN = Glud1 PE = 1 SV = 2	2	2	11,422	2047	WL	W
P04906	Glutathione S-transferase P OS = Rattus norvegicus GN = Gstp1 PE = 1 SV = 2	2	2	16,867	1545	W	WL
O35077	Glycerol-3-phosphate dehydrogenase [NAD(+)], cytoplasmic OS = Rattus norvegicus GN = Gpd1 PE = 1 SV = 4	7	6	70,467	1685	WL	W
P53534	Glycogen phosphorylase, brain form (Fragment) OS = Rattus norvegicus GN = Pygb PE = 1 SV = 3	10	3	115,182	1599	W	WL
P09811	Glycogen phosphorylase, liver form OS = Rattus norvegicus GN = Pygl PE = 1 SV = 5	11	5	89,307	1381	W	WL
P09812	Glycogen phosphorylase, muscle form OS = Rattus norvegicus GN = Pygm PE = 1 SV = 5	28	20	366,512	1212	W	WL
P55063	Heat shock 70 kDa protein 1-like OS = Rattus norvegicus GN = Hspa1l PE = 2 SV = 2	4	2	23,730	4650	W	WL
P0DMW0; P0DMW1	Heat shock 70 kDa protein 1A OS = Rattus norvegicus GN = Hspa1a PE = 2 SV = 1	5	4	49,142	2268	WL	W
P14659	Heat shock-related 70 kDa protein 2 OS = Rattus norvegicus GN = Hspa2 PE = 1 SV = 2	6	2	36,116	3054	WL	W
P01946	Hemoglobin subunit alpha-1/2 OS = Rattus norvegicus GN = Hba1 PE = 1 SV = 3	4	4	53,375	>5	WL	W
P02091	Hemoglobin subunit beta-1 OS = Rattus norvegicus GN = Hbb PE = 1 SV = 3	10	5	100,129	1641	WL	W
Q99NA5	Isocitrate dehydrogenase [NAD] subunit alpha, mitochondrial OS = Rattus norvegicus GN = Idh3a PE = 1 SV = 1	4	3	34,865	2031	W	WL
P56574	Isocitrate dehydrogenase [NADP], mitochondrial OS = Rattus norvegicus GN = Idh2 PE = 1 SV = 2	9	9	90,467	2444	WL	W
P04642	L-lactate dehydrogenase A chain OS = Rattus norvegicus GN = Ldha PE = 1 SV = 1	19	15	217,151	1368	WL	W
P42123	L-lactate dehydrogenase B chain OS = Rattus norvegicus GN = Ldhb PE = 1 SV = 2	2	2	23,421	>5	W	WL
P15650	Long-chain specific acyl-CoA dehydrogenase, mitochondrial OS = Rattus norvegicus GN = Acadl PE = 1 SV = 1	7	7	67,998	1183	WL	W
O88989	Malate dehydrogenase, cytoplasmic OS = Rattus norvegicus GN = Mdh1 PE = 1 SV = 3	8	8	91,019	>5	WL	W

P04636	Malate dehydrogenase, mitochondrial OS = Rattus norvegicus GN = Mdh2 PE = 1 SV = 2	10	10	135,689	1923	WL	W
P08503	Medium-chain specific acyl-CoA dehydrogenase, mitochondrial OS = Rattus norvegicus GN = Acadm PE = 1 SV = 1	5	5	43,799	>5	WL	W
Q3KR86	MICOS complex subunit Mic60 (Fragment) OS = Rattus norvegicus GN = Immt PE = 1 SV = 1	5	5	25,747	1943	W	WL
Q8R431	Monoglyceride lipase OS = Rattus norvegicus GN = Mgll PE = 1 SV = 1	2	2	9990	>5	WL	W
P02600	Myosin light chain 1/3, skeletal muscle isoform OS = Rattus norvegicus GN = Myl1 PE = 1 SV = 2	6	6	54,225	1740	WL	W
P04466	Myosin regulatory light chain 2, skeletal muscle isoform OS = Rattus norvegicus GN = Mylpf PE = 1 SV = 2	8	8	64,391	2438	W	WL
Q66HF1	NADH-ubiquinone oxidoreductase 75 kDa subunit, mitochondrial OS = Rattus norvegicus GN = Ndufs1 PE = 1 SV = 1	12	12	97,211	>5	WL	W
P83860	Orexigenic neuropeptide QRFP OS = Rattus norvegicus GN = Qrfp PE = 1 SV = 1	3	3	13,702	1250	W	WL
P02625	Parvalbumin alpha OS = Rattus norvegicus GN = Pvalb PE = 1 SV = 2	13	13	152,758	1991	W	WL
Q9R063	Peroxiredoxin-5, mitochondrial OS = Rattus norvegicus GN = Prdx5 PE = 1 SV = 1	2	2	25,977	>5	WL	W
P31044	Phosphatidylethanolamine-binding protein 1 OS = Rattus norvegicus GN = Pebp1 PE = 1 SV = 3	6	6	85,883	1290	WL	W
P38652	Phosphoglucomutase-1 OS = Rattus norvegicus GN = Pgm1 PE = 1 SV = 2	12	12	103,616	1971	WL	W
P16617	Phosphoglycerate kinase 1 OS = Rattus norvegicus GN = Pgk1 PE = 1 SV = 2	13	13	136,560	1621	WL	W
P16290	Phosphoglycerate mutase 2 OS = Rattus norvegicus GN = Pgam2 PE = 1 SV = 2	9	7	104,788	1395	WL	W
P0CG51; P62982; P62986; Q63429	Polyubiquitin-B OS = Rattus norvegicus GN = Ubb PE = 1 SV = 1	2	2	11,745	>5	W	WL
O88767	Protein DJ-1 OS = Rattus norvegicus GN = Park7 PE = 1 SV = 1	6	6	60,838	3753	W	WL
P49432	Pyruvate dehydrogenase E1 component subunit beta, mitochondrial OS = Rattus norvegicus GN = Pdhb PE = 1 SV = 2	5	4	37,396	>5	WL	W
P11980	Pyruvate kinase PKM OS = Rattus norvegicus GN = Pkm PE = 1 SV = 3	19	14	238,305	2324	W	WL
Q99P74	Ras-related protein Rab-27B OS = Rattus norvegicus GN = Rab27b PE = 2 SV = 3	2	2	11,728	>5	WL	W
P02770	Serum albumin OS = Rattus norvegicus GN = Alb PE = 1 SV = 2	11	11	105,678	2815	WL	W

P15651	Short-chain specific acyl-CoA dehydrogenase, mitochondrial OS = Rattus norvegicus GN = Acads PE = 1 SV = 2	4	4	20,323	1175	WL	W
P48721	Stress-70 protein, mitochondrial OS = Rattus norvegicus GN = Hspa9 PE = 1 SV = 3	7	7	42,165	>5	WL	W
Q920L2	Succinate dehydrogenase [ubiquinone] flavoprotein subunit, mitochondrial OS = Rattus norvegicus GN = Sdha PE = 1 SV = 1	2	2	12,812	3386	WL	W
B2GV06	Succinyl-CoA:3-ketoacid coenzyme A transferase 1, mitochondrial OS = Rattus norvegicus GN = Oxct1 PE = 1 SV = 1	3	3	21,939	1577	WL	W
Q9Z0V6	Thioredoxin-dependent peroxide reductase, mitochondrial OS = Rattus norvegicus GN = Prdx3 PE = 1 SV = 2	2	2	17,588	1459	WL	W
Q64428	Trifunctional enzyme subunit alpha, mitochondrial OS = Rattus norvegicus GN = Hadha PE = 1 SV = 2	7	7	51,229	1683	WL	W
Q60587	Trifunctional enzyme subunit beta, mitochondrial OS = Rattus norvegicus GN = Hadhb PE = 1 SV = 1	5	5	30,481	2139	WL	W
P48500	Triosephosphate isomerase OS = Rattus norvegicus GN = Tpi1 PE = 1 SV = 2	6	6	84,978	1278	WL	W
A0JPQ4	Tripartite motif-containing protein 72 OS = Rattus norvegicus GN = Trim72 PE = 1 SV = 1	8	8	64,513	>5	W	WL
P45953	Very long-chain specific acyl-CoA dehydrogenase, mitochondrial OS = Rattus norvegicus GN = Acadvl PE = 1 SV = 1	5	5	46,820	3580	WL	W
Q9Z2L0	Voltage-dependent anion-selective channel protein 1 OS = Rattus norvegicus GN = Vdac1 PE = 1 SV = 4	10	9	129,518	2695	W	WL
P81155	Voltage-dependent anion-selective channel protein 2 OS = Rattus norvegicus GN = Vdac2 PE = 1 SV = 2	4	3	45,846	>5	WL	W
Q9R1Z0	Voltage-dependent anion-selective channel protein 3 OS = Rattus norvegicus GN = Vdac3 PE = 1 SV = 2	8	4	80,447	4491	W	WL

Rats were distributed into Walker tumour-bearing (W) and Walker tumour-bearing fed a leucine-rich diet (WL) euthanazied at 21st day of the experiment. Statistical analysis described in Methods, showing the significant value for $p < 0.05$.

Table S8. Comparison of the highest and lowest proteins concentration identified in muscle of rats from C and W groups.

Accession	Description	Peptide Count	Unique Peptides	Confidence Score	Max Fold Change	Highest Mean Condition	Lowest Mean Condition
P62260; P63102; P68255; P68511	14-3-3 protein epsilon OS = Rattus norvegicus GN = Ywhae PE = 1 SV = 1	6	4	50,412	2535	C	W
Q5XI78	2-oxoglutarate dehydrogenase, mitochondrial OS = Rattus norvegicus GN = Ogdh PE = 1 SV = 1	13	12	100,131	4518	C	W
P17764	Acetyl-CoA acetyltransferase, mitochondrial OS = Rattus norvegicus GN = Acat1 PE = 1 SV = 1	6	5	50,816	>5	C	W
Q9ER34	Aconitate hydratase, mitochondrial OS = Rattus norvegicus GN = Aco2 PE = 1 SV = 2	17	17	164,364	>5	C	W
P39069	Adenylate kinase isoenzyme 1 OS = Rattus norvegicus GN = Ak1 PE = 1 SV = 3	8	8	81,599	>5	C	W
Q05962; Q09073	ADP/ATP translocase 1 OS = Rattus norvegicus GN = Slc25a4 PE = 1 SV = 3	14	14	144,973	>5	C	W
P04764	Alpha-enolase OS = Rattus norvegicus GN = Eno1 PE = 1 SV = 4	6	4	83,750	>5	C	W
P48037	Annexin A6 OS = Rattus norvegicus GN = Anxa6 PE = 1 SV = 2	10	10	65,081	>5	C	W
P00507	Aspartate aminotransferase, mitochondrial OS = Rattus norvegicus GN = Got2 PE = 1 SV = 2	8	8	66,125	4053	C	W
P15999	ATP synthase subunit alpha, mitochondrial OS = Rattus norvegicus GN = Atp5a1 PE = 1 SV = 2	18	17	215,323	2238	C	W
P10719	ATP synthase subunit beta, mitochondrial OS = Rattus norvegicus GN = Atp5b PE = 1 SV = 2	30	30	423,286	>5	C	W
Q06647	ATP synthase subunit O, mitochondrial OS = Rattus norvegicus GN = Atp5o PE = 1 SV = 1	6	6	59,574	>5	C	W
P47858	ATP-dependent 6-phosphofructokinase, muscle type OS = Rattus norvegicus GN = Pfkm PE = 1 SV = 3	21	21	196,870	1322	W	C
P15429	Beta-enolase OS = Rattus norvegicus GN = Eno3 PE = 1 SV = 3	17	14	303,239	>5	C	W
P19633	Calsequestrin-1 OS = Rattus norvegicus GN = Casq1 PE = 1 SV = 2	8	8	102,983	3589	C	W
P14141	Carbonic anhydrase 3 OS = Rattus norvegicus GN = Ca3 PE = 1 SV = 3	21	21	230,247	>5	C	W
Q8VHF5	Citrate synthase, mitochondrial OS = Rattus norvegicus GN = Cs PE = 1 SV = 1	9	9	59,154	>5	C	W

P00564	Creatine kinase M-type OS = Rattus norvegicus GN = Ckm PE = 1 SV = 2	27	27	301,125	2757	W	C
P09605	Creatine kinase S-type, mitochondrial OS = Rattus norvegicus GN = Ckmt2 PE = 1 SV = 2	10	9	124,786	3242	C	W
P32551	Cytochrome b-C complex subunit 2, mitochondrial OS = Rattus norvegicus GN = Uqcrc2 PE = 1 SV = 2	8	8	74,999	1440	C	W
P62898; P10715	Cytochrome c, somatic OS = Rattus norvegicus GN = Cycs PE = 1 SV = 2	7	7	63,065	>5	C	W
Q01205	Dihydrolipoyllysine-residue succinyltransferase component of 2-oxoglutarate dehydrogenase complex, mitochondrial OS = Rattus norvegicus GN = Dlst PE = 1 SV = 2	9	9	65,558	>5	C	W
P13803	Electron transfer flavoprotein subunit alpha, mitochondrial OS = Rattus norvegicus GN = Etfa PE = 1 SV = 4	7	7	57,949	>5	C	W
P62632	Elongation factor 1-alpha 2 OS = Rattus norvegicus GN = Eef1a2 PE = 1 SV = 1	7	7	66,018	>5	C	W
P05197	Elongation factor 2 OS = Rattus norvegicus GN = Eef2 PE = 1 SV = 4	7	7	52,980	>5	C	W
P05065; P09117	Fructose-bisphosphate aldolase A OS = Rattus norvegicus GN = Aldoa PE = 1 SV = 2	18	17	228,947	1476	C	W
P14408	Fumarate hydratase, mitochondrial OS = Rattus norvegicus GN = Fh PE = 1 SV = 1	6	6	52,473	>5	C	W
P07323	Gamma-enolase OS = Rattus norvegicus GN = Eno2 PE = 1 SV = 2	6	3	62,820	>5	C	W
Q6P6V0	Glucose-6-phosphate isomerase OS = Rattus norvegicus GN = Gpi PE = 1 SV = 1	16	16	170,441	>5	C	W
P04797	Glyceraldehyde-3-phosphate dehydrogenase OS = Rattus norvegicus GN = Gapdh PE = 1 SV = 3	28	28	376,187	>5	C	W
O35077	Glycerol-3-phosphate dehydrogenase [NAD(+)], cytoplasmic OS = Rattus norvegicus GN = Gpd1 PE = 1 SV = 4	7	7	56,540	>5	C	W
P53534	Glycogen phosphorylase, brain form (Fragment) OS = Rattus norvegicus GN = Pygb PE = 1 SV = 3	14	6	159,271	1518	C	W
P09812	Glycogen phosphorylase, muscle form OS = Rattus norvegicus GN = Pygm PE = 1 SV = 5	29	20	393,199	>5	W	C
P97541	Heat shock protein beta-6 OS = Rattus norvegicus GN = Hspb6 PE = 1 SV = 1	5	5	56,195	>5	C	W
P34058	Heat shock protein HSP 90-beta OS = Rattus norvegicus GN = Hsp90ab1 PE = 1 SV = 4	10	7	87,461	>5	C	W
P01946	Hemoglobin subunit alpha-1/2 OS = Rattus norvegicus GN = Hba1 PE = 1 SV = 3	5	5	70,058	>5	C	W

P02091	Hemoglobin subunit beta-1 OS = Rattus norvegicus GN = Hbb PE = 1 SV = 3	8	3	81,594	>5	C	W
P56574	Isocitrate dehydrogenase [NADP], mitochondrial OS = Rattus norvegicus GN = Idh2 PE = 1 SV = 2	8	8	100,563	>5	C	W
P04642; P19629	L-lactate dehydrogenase A chain OS = Rattus norvegicus GN = Ldha PE = 1 SV = 1	18	18	236,265	>5	C	W
P42123	L-lactate dehydrogenase B chain OS = Rattus norvegicus GN = Ldhb PE = 1 SV = 2	10	10	123,174	>5	C	W
O88989	Malate dehydrogenase, cytoplasmic OS = Rattus norvegicus GN = Mdh1 PE = 1 SV = 3	10	10	117,985	>5	C	W
P04636	Malate dehydrogenase, mitochondrial OS = Rattus norvegicus GN = Mdh2 PE = 1 SV = 2	9	9	97,959	>5	C	W
Q9QZ76	Myoglobin OS = Rattus norvegicus GN = Mb PE = 1 SV = 3	11	10	135,669	>5	C	W
P02600	Myosin light chain 1/3, skeletal muscle isoform OS = Rattus norvegicus GN = Myl1 PE = 1 SV = 2	7	7	59,360	>5	C	W
P04466	Myosin regulatory light chain 2, skeletal muscle isoform OS = Rattus norvegicus GN = Mylpf PE = 1 SV = 2	4	4	55,709	2792	C	W
P08733	Myosin regulatory light chain 2, ventricular/cardiac muscle isoform OS = Rattus norvegicus GN = Myl2 PE = 1 SV = 2	10	10	137,343	>5	C	W
Q641Y2	NADH dehydrogenase [ubiquinone] iron-sulfur protein 2, mitochondrial OS = Rattus norvegicus GN = Ndufs2 PE = 1 SV = 1	8	8	50,288	>5	C	W
Q66HF1	NADH-ubiquinone oxidoreductase 75 kDa subunit, mitochondrial OS = Rattus norvegicus GN = Ndufs1 PE = 1 SV = 1	9	9	68,609	>5	C	W
P38652	Phosphoglucomutase-1 OS = Rattus norvegicus GN = Pgm1 PE = 1 SV = 2	15	15	139,785	3449	C	W
P16617	Phosphoglycerate kinase 1 OS = Rattus norvegicus GN = Pgk1 PE = 1 SV = 2	19	19	228,293	1556	C	W
P25113	Phosphoglycerate mutase 1 OS = Rattus norvegicus GN = Pgam1 PE = 1 SV = 4	5	2	60,767	>5	C	W
P16290	Phosphoglycerate mutase 2 OS = Rattus norvegicus GN = Pgam2 PE = 1 SV = 2	8	5	124,675	3625	C	W
O88767	Protein DJ-1 OS = Rattus norvegicus GN = Park7 PE = 1 SV = 1	6	6	72,034	2218	C	W
P49432	Pyruvate dehydrogenase E1 component subunit beta, mitochondrial OS = Rattus norvegicus GN = Pdhb PE = 1 SV = 2	6	6	54,418	>5	C	W
P11980	Pyruvate kinase PKM OS = Rattus norvegicus GN = Pkm PE = 1 SV = 3	20	19	248,322	1914	W	C

Q64578	Sarcoplasmic/endoplasmic reticulum calcium ATPase 1 OS = Rattus norvegicus GN = Atp2a1 PE = 1 SV = 1	37	17	414,463	1566	C	W
P11507	Sarcoplasmic/endoplasmic reticulum calcium ATPase 2 OS = Rattus norvegicus GN = Atp2a2 PE = 1 SV = 1	27	11	284,470	1678	C	W
P18596	Sarcoplasmic/endoplasmic reticulum calcium ATPase 3 OS = Rattus norvegicus GN = Atp2a3 PE = 1 SV = 2	9	2	115,962	>5	W	C
P12346	Serotransferrin OS = Rattus norvegicus GN = Tf PE = 1 SV = 3	7	7	57,572	>5	C	W
P02770	Serum albumin OS = Rattus norvegicus GN = Alb PE = 1 SV = 2	14	14	168,502	>5	C	W
B2GV06	Succinyl-CoA:3-ketoacid coenzyme A transferase 1, mitochondrial OS = Rattus norvegicus GN = Oxct1 PE = 1 SV = 1	7	7	59,646	4540	C	W
Q64428	Trifunctional enzyme subunit alpha, mitochondrial OS = Rattus norvegicus GN = Hadha PE = 1 SV = 2	8	8	66,127	>5	C	W
P48500	Triosephosphate isomerase OS = Rattus norvegicus GN = Tpi1 PE = 1 SV = 2	7	7	94,512	4468	C	W
A0JPQ4	Tripartite motif-containing protein 72 OS = Rattus norvegicus GN = Trim72 PE = 1 SV = 1	11	11	80,236	>5	C	W
P45953	Very long-chain specific acyl-CoA dehydrogenase, mitochondrial OS = Rattus norvegicus GN = Acadvl PE = 1 SV = 1	7	7	52,646	>5	C	W
Q9Z2L0	Voltage-dependent anion-selective channel protein 1 OS = Rattus norvegicus GN = VdaC PE = 1 SV = 4	11	11	142,757	>5	W	C

Rats were distributed into control group (C) and Walker tumour-bearing group (W) euthanazized at 21st day of the experiment. Statistical analysis described in Methods, showing the significant value for $p < 0.05$.

Table S9. Comparison of the highest and lowest proteins concentration identified in muscle of rats from C and WL groups.

Accession	Description	Peptide Count	Unique Peptides	Confidence Score	Max Fold Change	Highest Mean Condition	Lowest Mean Condition
P62260; P63102; P68255; P68511	14-3-3 protein epsilon OS = Rattus norvegicus GN = Ywhae PE = 1 SV = 1	5	3	52,508	>5	C	WL
Q5XI78	2-oxoglutarate dehydrogenase, mitochondrial OS = Rattus norvegicus GN = Ogdh PE = 1 SV = 1	9	8	75,349	>5	C	WL
Q9ER34	Aconitate hydratase, mitochondrial OS = Rattus norvegicus GN = Aco2 PE = 1 SV = 2	21	21	194,599	1979	C	WL
P63259; P60711	Actin, cytoplasmic 2 OS = Rattus norvegicus GN = Actg1 PE = 1 SV = 1	7	2	54,187	1213	WL	C
P39069	Adenylate kinase isoenzyme 1 OS = Rattus norvegicus GN = Ak1 PE = 1 SV = 3	8	8	95,357	3572	C	WL
Q05962; Q09073	ADP/ATP translocase 1 OS = Rattus norvegicus GN = Slc25a4 PE = 1 SV = 3	14	14	143,809	>5	C	WL
P04764	Alpha-enolase OS = Rattus norvegicus GN = Eno1 PE = 1 SV = 4	7	5	92,358	>5	C	WL
P48037	Annexin A6 OS = Rattus norvegicus GN = Anxa6 PE = 1 SV = 2	11	11	69,944	>5	WL	C
P00507	Aspartate aminotransferase, mitochondrial OS = Rattus norvegicus GN = Got2 PE = 1 SV = 2	8	8	73,018	3030	C	WL
P15999	ATP synthase subunit alpha, mitochondrial OS = Rattus norvegicus GN = Atp5a1 PE = 1 SV = 2	16	14	202,195	1623	WL	C
P10719	ATP synthase subunit beta, mitochondrial OS = Rattus norvegicus GN = Atp5b PE = 1 SV = 2	28	28	404,595	>5	C	WL
Q06647	ATP synthase subunit O, mitochondrial OS = Rattus norvegicus GN = Atp5o PE = 1 SV = 1	7	7	65,907	2563	C	WL
P15429	Beta-enolase OS = Rattus norvegicus GN = Eno3 PE = 1 SV = 3	17	14	278,102	>5	C	WL
P19633	Calsequestrin-1 OS = Rattus norvegicus GN = Casq1 PE = 1 SV = 2	11	11	120,993	4486	C	WL
P14141	Carbonic anhydrase 3 OS = Rattus norvegicus GN = Ca3 PE = 1 SV = 3	21	21	222,582	>5	C	WL
Q8VHF5	Citrate synthase, mitochondrial OS = Rattus norvegicus GN = Cs PE = 1 SV = 1	8	8	64,380	>5	C	WL
P00564	Creatine kinase M-type OS = Rattus norvegicus GN = Ckm PE = 1 SV = 2	25	24	277,165	2881	WL	C
P09605	Creatine kinase S-type, mitochondrial OS = Rattus norvegicus GN = Ckmt2 PE = 1 SV = 2	7	7	96,218	3345	C	WL
P62898; P10715	Cytochrome c, somatic OS = Rattus norvegicus GN = Cycs PE = 1 SV = 2	7	7	69,649	>5	C	WL
P08461	Dihydrolipoyllysine-residue acetyltransferase component of pyruvate dehydrogenase complex, mitochondrial OS = Rattus norvegicus GN = Dlat PE = 1 SV = 3	12	11	73,384	1389	WL	C

P13803	Electron transfer flavoprotein subunit alpha, mitochondrial OS = Rattus norvegicus GN = Etfa PE = 1 SV = 4	8	8	72,038	>5	C	WL
P62632	Elongation factor 1-alpha 2 OS = Rattus norvegicus GN = Eef1a2 PE = 1 SV = 1	10	10	93,220	2919	WL	C
P05197	Elongation factor 2 OS = Rattus norvegicus GN = Eef2 PE = 1 SV = 4	7	7	52,980	2530	C	WL
P07483	Fatty acid-binding protein, heart OS = Rattus norvegicus GN = Fabp3 PE = 1 SV = 2	5	5	61,220	4733	C	WL
P05065	Fructose-bisphosphate aldolase A OS = Rattus norvegicus GN = Aldoa PE = 1 SV = 2	15	15	198,571	1335	C	WL
P14408	Fumarate hydratase, mitochondrial OS = Rattus norvegicus GN = Fh PE = 1 SV = 1	5	5	53,715	>5	C	WL
Q6P6V0	Glucose-6-phosphate isomerase OS = Rattus norvegicus GN = Gpi PE = 1 SV = 1	18	17	182,151	>5	C	WL
P04797; Q9ESV6	Glyceraldehyde-3-phosphate dehydrogenase OS = Rattus norvegicus GN = Gapdh PE = 1 SV = 3	28	28	334,619	>5	C	WL
O35077	Glycerol-3-phosphate dehydrogenase [NAD(+)], cytoplasmic OS = Rattus norvegicus GN = Gpd1 PE = 1 SV = 4	10	10	80,128	>5	C	WL
P53534	Glycogen phosphorylase, brain form (Fragment) OS = Rattus norvegicus GN = Pygb PE = 1 SV = 3	15	9	145,732	2454	WL	C
P09812	Glycogen phosphorylase, muscle form OS = Rattus norvegicus GN = Pygm PE = 1 SV = 5	28	21	371,008	4966	WL	C
P63018; P14659	Heat shock cognate 71 kDa protein OS = Rattus norvegicus GN = Hspa8 PE = 1 SV = 1	15	15	121,140	2320	WL	C
P82995	Heat shock protein HSP 90-alpha OS = Rattus norvegicus GN = Hsp90aa1 PE = 1 SV = 3	9	4	59,703	>5	C	WL
P01946	Hemoglobin subunit alpha-1/2 OS = Rattus norvegicus GN = Hba1 PE = 1 SV = 3	5	5	68,687	>5	C	WL
P11517	Hemoglobin subunit beta-2 OS = Rattus norvegicus PE = 1 SV = 2	9	2	92,041	>5	C	WL
P56574	Isocitrate dehydrogenase [NADP], mitochondrial OS = Rattus norvegicus GN = Idh2 PE = 1 SV = 2	10	10	110,383	>5	C	WL
P04642; P19629	L-lactate dehydrogenase A chain OS = Rattus norvegicus GN = Ldha PE = 1 SV = 1	18	17	243,105	>5	C	WL
P42123	L-lactate dehydrogenase B chain OS = Rattus norvegicus GN = Ldhb PE = 1 SV = 2	11	11	129,288	>5	C	WL
O88989	Malate dehydrogenase, cytoplasmic OS = Rattus norvegicus GN = Mdh1 PE = 1 SV = 3	11	11	129,953	>5	C	WL
P04636	Malate dehydrogenase, mitochondrial OS = Rattus norvegicus GN = Mdh2 PE = 1 SV = 2	12	12	121,041	2221	C	WL
Q9QZ76	Myoglobin OS = Rattus norvegicus GN = Mb PE = 1 SV = 3	10	10	137,123	>5	C	WL
P02600	Myosin light chain 1/3, skeletal muscle isoform OS = Rattus norvegicus GN = Myl1 PE = 1 SV = 2	7	7	65,838	>5	WL	C
P04466	Myosin regulatory light chain 2, skeletal muscle isoform OS = Rattus norvegicus GN = Mylpf PE = 1 SV = 2	6	6	69,892	4991	C	WL

P08733	Myosin regulatory light chain 2, ventricular/cardiac muscle isoform OS = Rattus norvegicus GN = Myl2 PE = 1 SV = 2	9	9	114,371	>5	C	WL
Q66HF1	NADH-ubiquinone oxidoreductase 75 kDa subunit, mitochondrial OS = Rattus norvegicus GN = Ndufs1 PE = 1 SV = 1	11	11	73,362	>5	WL	C
P02625	Parvalbumin alpha OS = Rattus norvegicus GN = Pvalb PE = 1 SV = 2	16	16	183,687	3729	WL	C
P38652	Phosphoglucomutase-1 OS = Rattus norvegicus GN = Pgm1 PE = 1 SV = 2	17	17	159,653	2633	C	WL
P16617	Phosphoglycerate kinase 1 OS = Rattus norvegicus GN = Pgk1 PE = 1 SV = 2	17	17	205,501	1722	WL	C
P16290	Phosphoglycerate mutase 2 OS = Rattus norvegicus GN = Pgam2 PE = 1 SV = 2	11	9	153,572	4126	C	WL
O88767	Protein DJ-1 OS = Rattus norvegicus GN = Park7 PE = 1 SV = 1	5	5	65,514	1456	WL	C
P11980	Pyruvate kinase PKM OS = Rattus norvegicus GN = Pkm PE = 1 SV = 3	19	17	226,641	1978	WL	C
Q64578	Sarcoplasmic/endoplasmic reticulum calcium ATPase 1 OS = Rattus norvegicus GN = Atp2a1 PE = 1 SV = 1	38	20	409,597	1216	C	WL
P11507	Sarcoplasmic/endoplasmic reticulum calcium ATPase 2 OS = Rattus norvegicus GN = Atp2a2 PE = 1 SV = 1	24	10	253,991	1406	C	WL
P18596	Sarcoplasmic/endoplasmic reticulum calcium ATPase 3 OS = Rattus norvegicus GN = Atp2a3 PE = 1 SV = 2	8	2	98,280	>5	WL	C
P12346	Serotransferrin OS = Rattus norvegicus GN = Tf PE = 1 SV = 3	7	7	57,572	>5	C	WL
P02770	Serum albumin OS = Rattus norvegicus GN = Alb PE = 1 SV = 2	12	12	153,224	>5	C	WL
B2GV06	Succinyl-CoA:3-ketoacid coenzyme A transferase 1, mitochondrial OS = Rattus norvegicus GN = Oxct1 PE = 1 SV = 1	7	7	53,469	>5	C	WL
Q64428	Trifunctional enzyme subunit alpha, mitochondrial OS = Rattus norvegicus GN = Hadha PE = 1 SV = 2	8	8	72,466	>5	C	WL
P48500	Triosephosphate isomerase OS = Rattus norvegicus GN = Tpi1 PE = 1 SV = 2	6	6	88,490	3247	C	WL
A0JPQ4	Tripartite motif-containing protein 72 OS = Rattus norvegicus GN = Trim72 PE = 1 SV = 1	12	12	84,453	2425	C	WL
P45953	Very long-chain specific acyl-CoA dehydrogenase, mitochondrial OS = Rattus norvegicus GN = Acadvl PE = 1 SV = 1	9	9	64,533	2001	C	WL
Q9Z2L0	Voltage-dependent anion-selective channel protein 1 OS = Rattus norvegicus GN = VdaC PE = 1 SV = 4	12	12	158,956	1249	WL	C
Q9R1Z0	Voltage-dependent anion-selective channel protein 3 OS = Rattus norvegicus GN = Vdac3 PE = 1 SV = 2	8	7	68,352	1632	WL	C

Rats were distributed into control group (C) and Walker tumour-bearing fed Leucine-rich diet (WL) euthanatized at 21st day of the experiment. Statistical analysis described in Methods, showing the significant value for $p < 0.05$.

Table S10. Comparison of the highest and lowest proteins concentration identified in muscle of rats from C and L groups.

Accession	Description	Peptide Count	Unique Peptides	Confidence Score	Max Fold Change	Highest Mean Condition	Lowest Mean Condition
P62260; P63102; P68255	14-3-3 protein epsilon OS = Rattus norvegicus GN = Ywhae PE = 1 SV = 1	5	3	52,508	>5	L	C
Q5XI78	2-oxoglutarate dehydrogenase, mitochondrial OS = Rattus norvegicus GN = Ogdh PE = 1 SV = 1	9	9	69,186	>5	L	C
Q9ER34	Aconitate hydratase, mitochondrial OS = Rattus norvegicus GN = Aco2 PE = 1 SV = 2	22	22	203,808	1892	L	C
P39069	Adenylate kinase isoenzyme 1 OS = Rattus norvegicus GN = Ak1 PE = 1 SV = 3	8	8	89,245	>5	L	C
Q05962	ADP/ATP translocase 1 OS = Rattus norvegicus GN = Slc25a4 PE = 1 SV = 3	15	8	159,810	>5	L	C
P04764	Alpha-enolase OS = Rattus norvegicus GN = Eno1 PE = 1 SV = 4	8	4	91,247	>5	L	C
P48037	Annexin A6 OS = Rattus norvegicus GN = Anxa6 PE = 1 SV = 2	12	12	81,517	2776	L	C
P13221	Aspartate aminotransferase, cytoplasmic OS = Rattus norvegicus GN = Got1 PE = 1 SV = 3	12	12	124,160	1324	L	C
P00507	Aspartate aminotransferase, mitochondrial OS = Rattus norvegicus GN = Got2 PE = 1 SV = 2	9	8	79,141	>5	L	C
P15999	ATP synthase subunit alpha, mitochondrial OS = Rattus norvegicus GN = Atp5a1 PE = 1 SV = 2	20	19	238,291	1263	C	L
P10719	ATP synthase subunit beta, mitochondrial OS = Rattus norvegicus GN = Atp5b PE = 1 SV = 2	30	30	443,229	1835	L	C
Q06647	ATP synthase subunit O, mitochondrial OS = Rattus norvegicus GN = Atp5o PE = 1 SV = 1	7	7	64,487	>5	L	C
P15429	Beta-enolase OS = Rattus norvegicus GN = Eno3 PE = 1 SV = 3	21	15	292,836	>5	L	C
P19633	Calsequestrin-1 OS = Rattus norvegicus GN = Casq1 PE = 1 SV = 2	10	10	121,508	3672	L	C
P14141	Carbonic anhydrase 3 OS = Rattus norvegicus GN = Ca3 PE = 1 SV = 3	20	20	208,125	>5	L	C
Q8VHF5	Citrate synthase, mitochondrial OS = Rattus norvegicus GN = Cs PE = 1 SV = 1	9	9	79,713	3617	L	C
P00564	Creatine kinase M-type OS = Rattus norvegicus GN = Ckm PE = 1 SV = 2	27	26	261,262	1742	C	L
P09605	Creatine kinase S-type, mitochondrial OS = Rattus norvegicus GN = Ckmt2 PE = 1 SV = 2	8	8	102,098	3087	L	C
P32551	Cytochrome b-C complex subunit 2, mitochondrial OS = Rattus norvegicus GN = Uqcrc2 PE = 1 SV = 2	11	11	113,137	1412	L	C

P62898; P10715	Cytochrome c, somatic OS = Rattus norvegicus GN = Cycs PE = 1 SV = 2	10	10	78,878	>5	L	C
Q01205	Dihydrolipoyllysine-residue succinyltransferase component of 2-oxoglutarate dehydrogenase complex, mitochondrial OS = Rattus norvegicus GN = Dlst PE = 1 SV = 2	8	6	57,546	1301	L	C
P13803	Electron transfer flavoprotein subunit alpha, mitochondrial OS = Rattus norvegicus GN = Etfa PE = 1 SV = 4	7	7	65,595	>5	L	C
P62632	Elongation factor 1-alpha 2 OS = Rattus norvegicus GN = Eef1a2 PE = 1 SV = 1	9	9	82,942	>5	L	C
P05197	Elongation factor 2 OS = Rattus norvegicus GN = Eef2 PE = 1 SV = 4	7	7	52,980	4041	L	C
P14604	Enoyl-CoA hydratase, mitochondrial OS = Rattus norvegicus GN = Echs1 PE = 1 SV = 1	5	5	51,696	1622	L	C
P07483	Fatty acid-binding protein, heart OS = Rattus norvegicus GN = Fabp3 PE = 1 SV = 2	4	4	54,742	4908	L	C
P05065	Fructose-bisphosphate aldolase A OS = Rattus norvegicus GN = Aldoa PE = 1 SV = 2	15	14	190,406	1656	L	C
P14408	Fumarate hydratase, mitochondrial OS = Rattus norvegicus GN = Fh PE = 1 SV = 1	6	6	59,659	>5	L	C
P47819	Glial fibrillary acidic protein OS = Rattus norvegicus GN = Gfap PE = 1 SV = 2	8	6	61,300	2130	L	C
Q6P6V0	Glucose-6-phosphate isomerase OS = Rattus norvegicus GN = Gpi PE = 1 SV = 1	16	16	182,123	>5	L	C
P04797	Glyceraldehyde-3-phosphate dehydrogenase OS = Rattus norvegicus GN = Gapdh PE = 1 SV = 3	30	30	372,758	4038	L	C
O35077	Glycerol-3-phosphate dehydrogenase [NAD(+)], cytoplasmic OS = Rattus norvegicus GN = Gpd1 PE = 1 SV = 4	7	7	70,296	>5	L	C
P53534	Glycogen phosphorylase, brain form (Fragment) OS = Rattus norvegicus GN = Pygb PE = 1 SV = 3	16	9	171,164	1979	C	L
P09811	Glycogen phosphorylase, liver form OS = Rattus norvegicus GN = Pygl PE = 1 SV = 5	6	2	79,120	>5	C	L
P09812	Glycogen phosphorylase, muscle form OS = Rattus norvegicus GN = Pygm PE = 1 SV = 5	28	21	412,041	4659	C	L
P63018; P14659	Heat shock cognate 71 kDa protein OS = Rattus norvegicus GN = Hspa8 PE = 1 SV = 1	17	16	133,626	1363	C	L
P97541	Heat shock protein beta-6 OS = Rattus norvegicus GN = Hspb6 PE = 1 SV = 1	5	5	64,890	>5	L	C
P82995	Heat shock protein HSP 90-alpha OS = Rattus norvegicus GN = Hsp90aa1 PE = 1 SV = 3	10	5	64,875	4651	L	C
P34058	Heat shock protein HSP 90-beta OS = Rattus norvegicus GN = Hsp90ab1 PE = 1 SV = 4	15	10	110,744	1225	L	C

P01946	Hemoglobin subunit alpha-1/2 OS = Rattus norvegicus GN = Hba1 PE = 1 SV = 3	5	5	71,021	>5	L	C
P02091	Hemoglobin subunit beta-1 OS = Rattus norvegicus GN = Hbb PE = 1 SV = 3	11	3	110,396	>5	L	C
P11517	Hemoglobin subunit beta-2 OS = Rattus norvegicus PE = 1 SV = 2	10	2	98,199	>5	L	C
P56574	Isocitrate dehydrogenase [NADP], mitochondrial OS = Rattus norvegicus GN = Idh2 PE = 1 SV = 2	13	13	140,357	>5	L	C
P04642; P19629	L-lactate dehydrogenase A chain OS = Rattus norvegicus GN = Ldha PE = 1 SV = 1	18	18	220,936	>5	L	C
P42123	L-lactate dehydrogenase B chain OS = Rattus norvegicus GN = Ldhb PE = 1 SV = 2	12	12	134,910	>5	L	C
O88989	Malate dehydrogenase, cytoplasmic OS = Rattus norvegicus GN = Mdh1 PE = 1 SV = 3	9	9	118,035	2826	L	C
P04636	Malate dehydrogenase, mitochondrial OS = Rattus norvegicus GN = Mdh2 PE = 1 SV = 2	10	10	125,356	3883	L	C
Q9QZ76	Myoglobin OS = Rattus norvegicus GN = Mb PE = 1 SV = 3	10	9	135,191	>5	L	C
P02600	Myosin light chain 1/3, skeletal muscle isoform OS = Rattus norvegicus GN = MyL PE = 1 SV = 2	10	10	76,614	2823	L	C
P04466	Myosin regulatory light chain 2, skeletal muscle isoform OS = Rattus norvegicus GN = Mylpf PE = 1 SV = 2	6	6	75,768	2790	L	C
P08733	Myosin regulatory light chain 2, ventricular/cardiac muscle isoform OS = Rattus norvegicus GN = Myl2 PE = 1 SV = 2	10	10	138,101	4893	L	C
Q66HF1	NADH-ubiquinone oxidoreductase 75 kDa subunit, mitochondrial OS = Rattus norvegicus GN = Ndufs1 PE = 1 SV = 1	13	13	83,490	1327	C	L
P38652	Phosphoglucomutase-1 OS = Rattus norvegicus GN = Pgm1 PE = 1 SV = 2	14	13	138,900	>5	L	C
P16617	Phosphoglycerate kinase 1 OS = Rattus norvegicus GN = Pgk1 PE = 1 SV = 2	19	18	239,126	1270	L	C
P25113	Phosphoglycerate mutase 1 OS = Rattus norvegicus GN = Pgam1 PE = 1 SV = 4	5	3	66,858	>5	L	C
P16290	Phosphoglycerate mutase 2 OS = Rattus norvegicus GN = Pgam2 PE = 1 SV = 2	11	9	161,995	>5	L	C
O88767	Protein DJ-1 OS = Rattus norvegicus GN = Park7 PE = 1 SV = 1	4	4	57,187	2148	L	C
P49432	Pyruvate dehydrogenase E1 component subunit beta, mitochondrial OS = Rattus norvegicus GN = Pdhb PE = 1 SV = 2	9	8	83,970	>5	L	C
P18596	Sarcoplasmic/endoplasmic reticulum calcium ATPase 3 OS = Rattus norvegicus GN = Atp2a3 PE = 1 SV = 2	12	5	120,046	2230	C	L
P12346	Serotransferrin OS = Rattus norvegicus GN = Tf PE = 1 SV = 3	7	7	57,572	>5	L	C
P02770	Serum albumin OS = Rattus norvegicus GN = Alb PE = 1 SV = 2	15	15	179,396	3214	L	C

B2GV06	Succinyl-CoA:3-ketoacid coenzyme A transferase 1, mitochondrial OS = Rattus norvegicus GN = Oxct1 PE = 1 SV = 1	7	7	59,646	>5	L	C
P46462	Transitional endoplasmic reticulum ATPase OS = Rattus norvegicus GN = Vcp PE = 1 SV = 3	16	16	109,910	2917	L	C
Q64428	Trifunctional enzyme subunit alpha, mitochondrial OS = Rattus norvegicus GN = Hadha PE = 1 SV = 2	8	8	66,836	>5	L	C
P48500	Triosephosphate isomerase OS = Rattus norvegicus GN = Tpi1 PE = 1 SV = 2	6	6	106,470	>5	L	C
A0JPQ4	Tripartite motif-containing protein 72 OS = Rattus norvegicus GN = Trim72 PE = 1 SV = 1	10	10	72,206	2112	L	C
P68370; Q6AYZ1; Q6P9V9	Tubulin alpha-1A chain OS = Rattus norvegicus GN = Tuba1a PE = 1 SV = 1	7	6	60,748	2573	C	L
P45953	Very long-chain specific acyl-CoA dehydrogenase, mitochondrial OS = Rattus norvegicus GN = Acadvl PE = 1 SV = 1	8	8	64,716	1496	L	C
Q9Z2L0	Voltage-dependent anion-selective channel protein 1 OS = Rattus norvegicus GN = VdaC PE = 1 SV = 4	12	11	161,059	1434	C	L
P81155	Voltage-dependent anion-selective channel protein 2 OS = Rattus norvegicus GN = Vdac2 PE = 1 SV = 2	5	4	57,398	1659	L	C
Q9R1Z0	Voltage-dependent anion-selective channel protein 3 OS = Rattus norvegicus GN = Vdac3 PE = 1 SV = 2	8	7	71,414	2925	L	C

Rats were distributed into control group (C) and leucine-rich diet (L) euthanatized at 21st day of the experiment. Statistical analysis described in Methods, showing the significant value for $p < 0.05$.

© 2020 by the authors. Licensee MDPI, Basel, Switzerland. This article is an open access article distributed under the terms and conditions of the Creative Commons Attribution (CC BY) license (http://creativecommons.org/licenses/by/4.0/).
